# Stage of Gestation at Porcine Epidemic Diarrhea Virus Infection of Pregnant Swine Impacts Maternal Immunity and Lactogenic Immune Protection of Neonatal Suckling Piglets

**DOI:** 10.3389/fimmu.2019.00727

**Published:** 2019-04-24

**Authors:** Stephanie N. Langel, Francine C. Paim, Moyasar A. Alhamo, Alexandra Buckley, Albert Van Geelen, Kelly M. Lager, Anastasia N. Vlasova, Linda J. Saif

**Affiliations:** ^1^Food Animal Health Research Program, Department of Veterinary Preventive Medicine, Ohio Agricultural Research and Development Center, College of Food, Agriculture and Environmental Sciences, College of Veterinary Medicine, The Ohio State University, Wooster, OH, United States; ^2^National Animal Disease Center, Agricultural Research Service, USDA, Ames, IA, United States

**Keywords:** swine, PEDV, pregnancy, lactogenic immunity, gut-mammary-secretory IgA axis

## Abstract

During pregnancy, the maternal immune response changes dramatically over the course of gestation. This has implications for generation of lactogenic immunity and subsequent protection in suckling neonates against enteric viral infections. For example, porcine epidemic diarrhea virus (PEDV) is an alphacoronavirus that causes acute diarrhea in neonatal piglets. Due to the high virulence of PEDV and the naïve, immature immune system of neonatal suckling piglets, passive lactogenic immunity to PEDV induced during pregnancy, via the gut-mammary gland (MG)-secretory IgA (sIgA) axis, is critical for piglet protection. However, the anti-PEDV immune response during pregnancy and stage of gestation required to optimally stimulate the gut-MG-sIgA axis is undefined. We hypothesize that there is a gestational window in which non-lethal PEDV infection of pregnant gilts influences maximum lymphocyte mucosal trafficking to the MG, resulting in optimal passive lactogenic protection in suckling piglets. To understand how the stages of gestation affect maternal immune responses to PEDV, three groups of gilts were orally infected with PEDV in the first, second or third trimester. Control (mock) gilts were inoculated with medium in the third trimester. To determine if lactogenic immunity correlated with protection, all piglets were PEDV-challenged at 3–5 days postpartum. PEDV infection of gilts at different stages of gestation significantly affected multiple maternal systemic immune parameters prepartum, including cytokines, B cells, PEDV antibodies (Abs), and PEDV antibody secreting cells (ASCs). Pregnant second trimester gilts had significantly higher levels of circulating PEDV IgA and IgG Abs and ASCs and PEDV virus neutralizing (VN) Abs post PEDV infection. Coinciding with the significantly higher PEDV Ab responses in second trimester gilts, the survival rate of their PEDV-challenged piglets was 100%, compared with 87.2, 55.9, and 5.7% for first, third, and mock litters, respectively. Additionally, piglet survival positively correlated with PEDV IgA Abs and ASCs and VN Abs in milk and PEDV IgA and IgG Abs in piglet serum. Our findings have implications for gestational timing of oral attenuated PEDV maternal vaccines, whereby PEDV intestinal infection in the second trimester optimally stimulated the gut-MG-sIgA axis resulting in 100% lactogenic immune protection in suckling piglets.

## Introduction

Diarrheal diseases in young animals account for an estimated multi-million dollar loss to the livestock industry annually due to the livestock industry annually due to mortality, reduced weight gain, treatment costs, and trade sanctions on exporting animal products from infected countries (1, 2). For example, porcine epidemic diarrhea virus (PEDV) is a highly virulent re-emerging enteric coronavirus that causes acute diarrhea, dehydration, and death in neonatal piglets (3). It has killed over 8.5 million piglets since its emergence in the US in 2013. In adult pigs, PEDV causes watery diarrhea, depression, and anorexia as well as agalactia and reduced reproductive performance (1–3). Lactogenic immunity remains the most promising and effective way to protect neonatal suckling piglets from enteric diseases like PEDV (4, 5). This is dependent on trafficking of pathogen-specific IgA^+^ plasmablasts to the mammary gland (MG) and accumulation of secretory IgA (sIgA) antibodies (Abs) in milk, defined as the gut-MG-sIgA axis (6–8). Understanding the regulation of mucosal homing receptor and chemokine expression is critical to generate sufficient lactogenic immunity for piglet protection. For example, chemokine receptor (CCR)10, a lymphocyte gut homing marker, is required for IgA^+^ plasmablast recruitment to the MG in mice and humans (9–11). Additionally, an increase in lymphocyte migration to the MG in swine at the end of gestation and during lactation coincides with an increase in α_4_β_7_ integrin, another lymphocyte gut homing marker, on B cells (12). Identifying factors that influence lymphocyte migration and the gut-MG-sIgA axis may lead to improved PEDV vaccine regimens in gestating swine, boosting overall herd immunity and health and industry productivity.

Maternal vaccination that increases the amount of passively transferred protective Abs in milk, induced via the gut-MG-sIgA axis, is the strategy used to protect suckling piglets from PEDV immediately after birth (4, 5). For example, in swine, high rates of protection against another porcine enteric alphacoronavirus, transmissible gastroenteritis virus (TGEV) in piglets is achieved when pregnant sows are orally infected with live virulent virus (5–7, 13–15). The increased rate of protection was associated with high titers of IgA Abs in colostrum and milk. This demonstrates that enteric viral infection stimulates the intestinal mucosa influencing lactogenic immunity via the gut-MG-sIgA axis (4, 5). This model system can be used in the context of PEDV, as similar maternal vaccination strategies are needed for initiation of the gut-MG-sIgA axis and piglet protection (4, 12, 16, 17). We showed previously in third trimester pregnant gilts that administering a higher dose of virulent PEDV increased virus neutralizing (VN) Ab titers in colostrum/milk and piglet protection compared with a lower dose (4). Despite this, field reports demonstrate incomplete and variable protection in orally PEDV-infected gestating swine (4). Furthermore, the optimal stage of gestation to initiate the gut-MG-sIgA axis by means of natural infection or oral vaccination in naïve pregnant swine to generate protective lactogenic immunity is unknown.

Pregnancy modulates immunological processes that change over the course of gestation (18). For example, during the first trimester of gestation, levels of innate and proinflammatory factors increase, facilitating embryo implantation (19, 20). As pregnancy progresses, inflammatory cytokines decrease and regulatory cells and cytokines increase to support fetal growth and development to prevent rejection of the fetus (21–24). In the third trimester of gestation, the immunoregulatory environment is retained until immediately prior to parturition when proinflammatory and tissue repair factors increase, promoting the contraction of the uterus and expulsion of the fetus and placenta (18). The ability of pregnancy to differentially modulate the immune response explains why the severity of illness and efficacy of vaccination is dependent on stage of gestation. For example, the risk and severity of influenza (25), malaria (26), and listeria (27) is higher for women in their third trimester than other gestational stages. Additionally, in women vaccinated with the trivalent inactivated influenza vaccine, seroconversion rates were higher in late third trimester compared with first trimester-vaccinated women (28). Due to the differences in immune responses at different stages of pregnancy, it is important to consider stage of gestation when designing vaccines aimed to increase lactogenic immunity and passive transfer of protective Abs in colostrum/milk from mother to neonate.

In this study, we infected pregnant first parity gilts in their first, second and third trimesters of gestation with PEDV to determine the impact of stage of gestation on generation of maternal B-cell immunity, the gut-MG-sIgA axis and lactogenic immune protection in PEDV challenged piglets. Our goal was to identify innate and adaptive immune factors during pregnancy that influence lymphocyte trafficking, in addition to immune correlates of lactogenic immune protection in neonatal suckling piglets. Understanding the impact of stage of gestation at PEDV infection or exposure on maternal immunity will allow more precise maternal vaccination protocols to target the time when the animal is most immunologically responsive. Optimizing vaccine efficacy for gestating and lactating animals will enhance lactogenic immunity in neonates and decrease morbidity and mortality associated with neonatal enteric disease.

## Results

### Overall Summary of Results and Significance for the Major Immune and PEDV Protection Parameters Assessed

**Figure 1:**
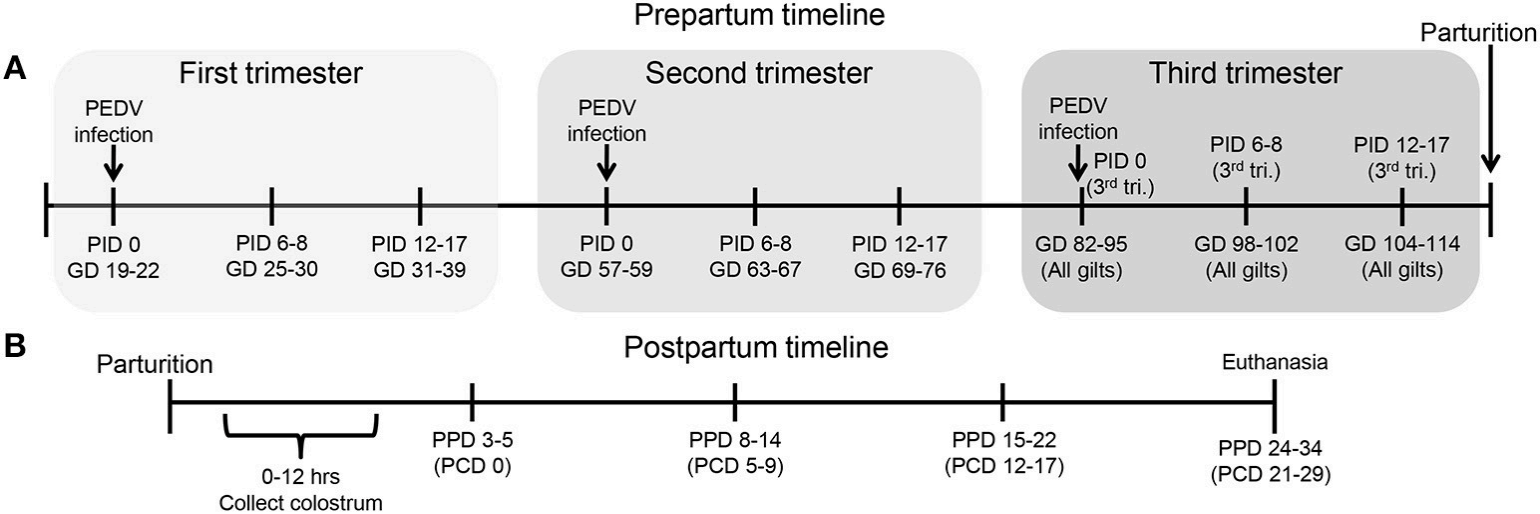
Schematic diagram of the experimental design showing gilt porcine epidemic diarrhea virus (PEDV) infection and sample time points at post infection day (PID) 0, 6–8, 12–17 and gestation day (GD) 82–95, 98–102, and 104–114, **(B)** piglet PEDV challenge at 3–5 postpartum day (PPD) and sample time points at PPD 3–5 (PCD [post challenge day] 0), PPD 8–14 (PCD 5–9), PPD 15–22 (PCD 12–17), and PPD 24–34 (PCD 21–29).

An overall summary of the statistically significant results (Table 1) illustrates that PEDV infection of gilts at different stages of gestation (Figure 1) affects multiple maternal systemic immune parameters prepartum, including natural killer (NK) cells, cytokines, B cells, and PEDV Abs and antibody secreting cells (ASCs). In addition, significant postpartum effects on lactogenic immune parameters in colostrum and milk were observed including significantly increased PEDV IgA (colostrum and milk) and IgG (colostrum only) ASCs and PEDV IgA Abs and VN Abs in PEDV-infected second trimester gilts (Table 1). Notably several gilt [PEDV IgA (colostrum and milk) and IgG (colostrum only) ASCs, Abs, and VN Abs] and piglet (serum PEDV IgA and IgG Abs) parameters were positively correlated with piglet survival rates (Table 2), demonstrating the association between IgA ASCs and Abs and VN Abs and lactogenic immune protection of suckling neonates. The detailed results for each parameter are described in the following sections.

**Table 1:** Overall summary of results and significance for the major immune and PEDV protection parameters assessed.

**Parameter**	**Result**	**Immune response, site**	**Significance**
**I. MATERNAL IMMUNITY (PREPARTUM)**			
Gilt blood natural killer (NK) cell frequency and activity	1st trimester gilts had significantly higher mean NK cell frequencies and cytotoxic activity	Innate, systemic	NK cell frequency/activity in blood may modulate PEDV specific B cell response in 1st trimester gilts
Gilt serum transforming growth factor (TGF)-β concentration	2nd trimester gilts had significantly higher mean concentrations of serum TGF-β	T regulatory cytokine, systemic	Elevated serum TGF-β in 2nd trimester gilts may enhance PEDV B cell/antibody (Ab) responses
Gilt blood α4^+^β7^+^ B cell frequency	2nd and 3rd trimester gilts had significantly higher mean frequencies of blood α4^+^β7^+^ B cells	B cell, systemic	Increased circulating frequencies of α4^+^β7^+^ B cells in 2nd/3rd trimester gilts may facilitate trafficking to the mammary gland
Gilt blood CD2^−^CD21^+^ B cell frequency	2nd and 3rd trimester gilts had significantly higher mean frequencies of blood CD2^−^CD21^+^ (activated and/or primed) B cells	B cell, systemic	Circulating frequencies of CD2^−^CD21^+^ B cells increase with gestational stage and may influence B cell activation/survival
Gilt blood PEDV IgA and IgG antibody secreting cells (ASCs)	2nd trimester gilts had significantly higher blood mean PEDV IgA and IgG ASCs	B cell, systemic	Increased blood PEDV IgA and IgG ASCs in 2nd trimester gilts is associated with ASC trafficking to the mammary gland
Gilt blood PEDV IgA, IgG, and virus neutralization (VN) Abs	2nd trimester gilts had significantly higher mean serum PEDV IgA, IgG and VN Abs	B cell/Abs, systemic	Increased serum PEDV IgA, IgG, and VN Ab in 2nd trimester gilts is associated with Ab accumulation in colostrum/milk
**II. LACTOGENIC IMMUNITY (POSTPARTUM)**			
Gilt colostrum/milk PEDV IgA and IgG ASCs	2nd trimester gilts had significantly higher mean PEDV IgA (in colostrum/milk) and IgG (in colostrum) ASCs	B cell, colostrum/milk	Increased PEDV IgA (colostrum/milk) and IgG ASCs (colostrum) in 2nd trimester gilts is correlated with lactogenic immune protection in piglets
Gilt colostrum/milk PEDV IgA and VN Abs	2nd trimester gilts had significantly higher mean colostrum/milk PEDV IgA and VN Abs	B cell/Abs, colostrum/milk	Increased colostrum/milk PEDV IgA and VN Abs in 2nd trimester gilts is correlated with lactogenic immune protection in piglets
**III. PIGLET PROTECTION (POSTPARTUM)**			
Piglet survival rate	2nd trimester litters had 100% survival rate	Piglet PEDV protection	Piglet survival rates were positively correlated with PEDV IgA ASCs and Abs and VN Abs in milk
Piglet PEDV RNA shedding titers and fecal consistency	2nd trimester litters had significantly lower mean PEDV RNA shedding titers and fecal consistency	Piglet PEDV protection, intestine	Increased lactogenic immune protection decreased viral shedding and diarrhea in 2nd trimester litters
Piglet serum IgA and IgG Abs	2nd trimester litters had significantly higher mean serum PEDV IgA and IgG Ab titers	B cell/Abs, systemic	Increased piglet serum IgA and IgG Abs positively correlated with survival rates

**Table 2:** Spearman's non-parametric correlations between piglet survival rate and numbers of PEDV IgA and IgG antibody secreting cells (ASCs) and log_10_-transformed PEDV IgA, IgG, and virus neutralizing (VN) antibody (Ab) titers in colostrum, postpartum day (PPD) 3–5 milk, PPD 8–14 milk, and PPD 15–22 milk in first, second, and third trimester PEDV-infected gilts.

		**PEDV IgA ASCs**	**PEDV IgG ASCs**	**PEDV IgA Abs**	**PEDV IgG Abs**	**PEDV VN Abs**
Colostrum	*r*	0.71	0.76	0.83	0.29	0.60
	*P*-value	0.0097	0.01	0.0005	0.33	0.03
PPD 3-5 milk	*r*	0.61	0.55	0.38	0.50	0.56
	*P*-value	0.04	0.051	0.22	0.10	0.06
PPD 8-14 milk	*r*	0.77	0.55	0.73	0.17	0.68
	*P*-value	0.0019	0.07	0.0067	0.59	0.02
PPD 12-22 milk	*r*	0.44	0.42	0.32	−0.50	0.61
	*P*-value	0.15	0.20	0.33	0.10	0.048

^*^P < 0.05, ^**^1P < 0.01, ^***^P < 0.001.

### Third Trimester Gilts Had Significantly Higher PEDV RNA Shedding Titers and More Severe PEDV-Induced Diarrhea

**Figure 2:**
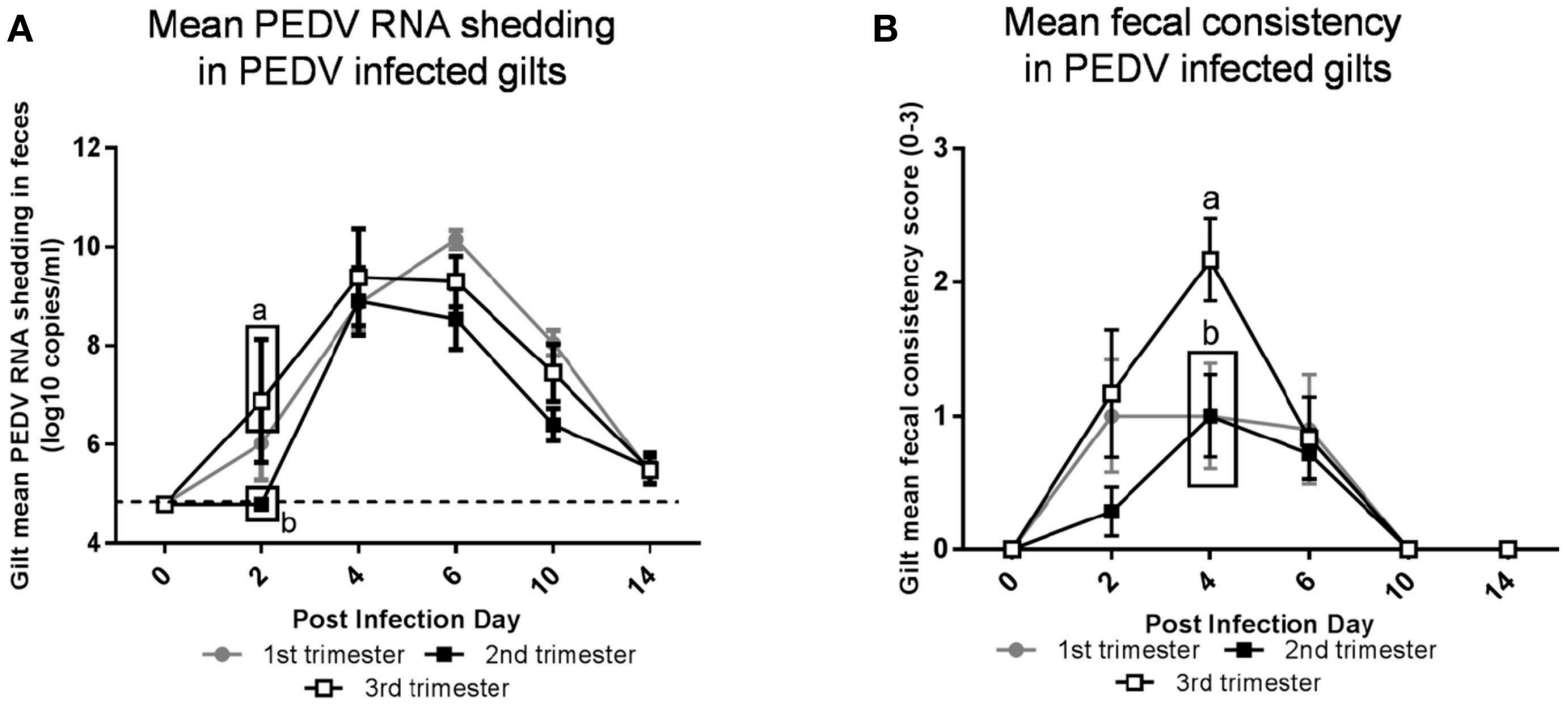
Third trimester gilts had significantly higher porcine epidemic diarrhea virus (PEDV) RNA shedding titers at post infection day (PID) 2 compared with second trimester gilts and significantly greater PEDV-induced diarrhea at PID 4 compared with first and second trimester gilts. **(A)** PEDV RNA shedding titers were determined by real time quantitative polymerase chain reaction (qRT-PCR) and expressed as log_10_ copies/ml. **(B)** Diarrhea was determined by fecal consistency score >1 (fecal consistency was scored as follows: 0, normal; 1, pasty/semiliquid; 2, liquid; 3, watery). PEDV RNA shedding titers and fecal consistency scores were measured at PID 0, 2, 4, 6, 10, and 14. Different letters indicate significant differences among treatment groups at the same time point (mean ± SEM). Statistical analysis was performed using the two-way ANOVA with repeated measures and Bonferroni's correction for multiple comparisons.

Third trimester gilts had significantly higher PEDV RNA shedding titers at PID 2 compared with second trimester gilts (Figure 2A). Additionally, second trimester gilts had delayed onset of PEDV RNA shedding compared with first and third trimester gilts. Fecal consistency scores at PID 4 were significantly higher in third compared with second and first trimester gilts and third trimester gilts were the only treatment group with clinical diarrhea (mean fecal consistency score of >1) (Figure 2B).

### Stage of Gestation Modulated Innate Immune Parameters

**Figure 3:**
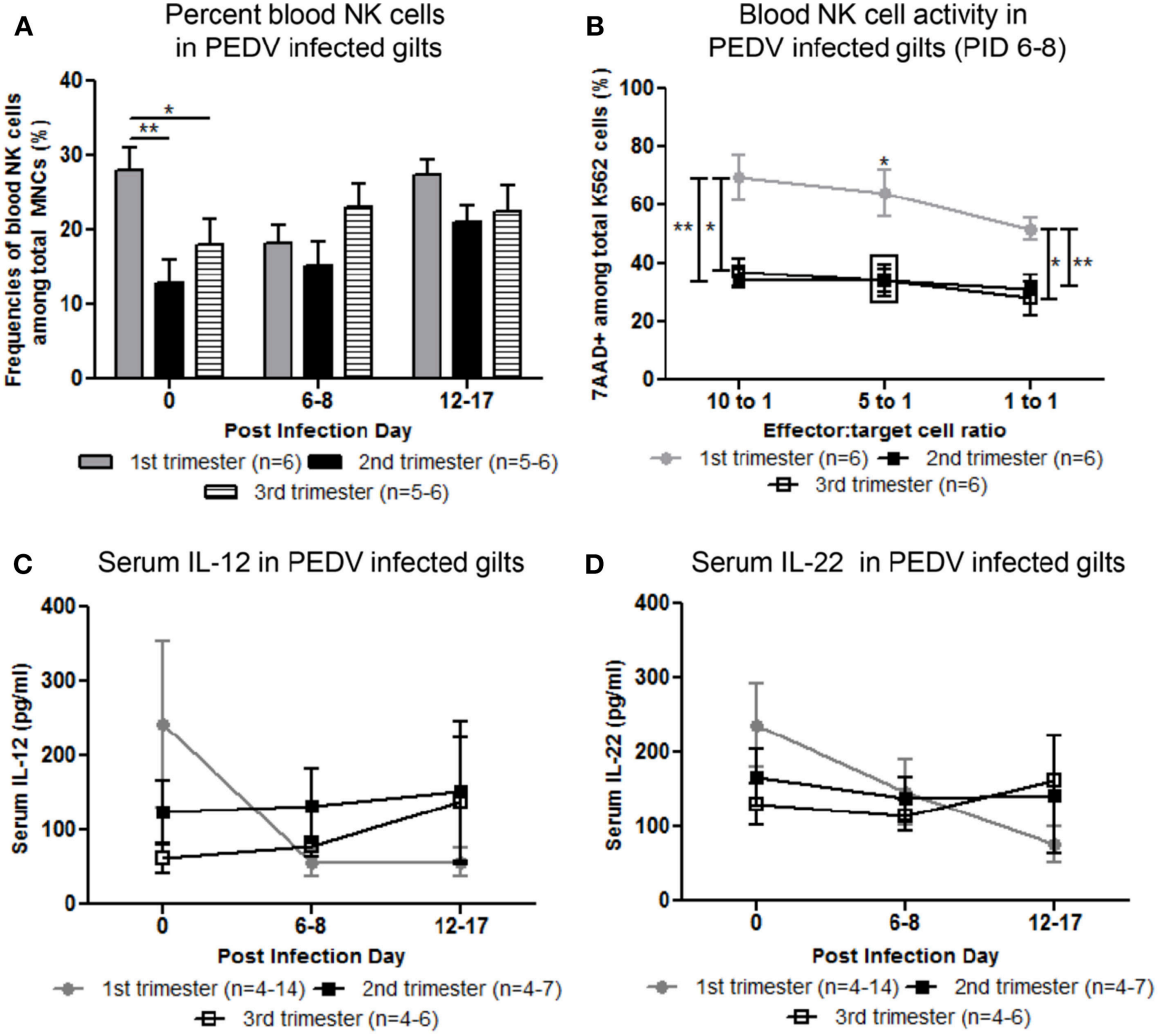
Peripheral natural killer (NK) cell frequency and function and serum interleukin (IL)-12 and IL-22 concentrations were increased in first compared with second and third trimester gilts. **(A)** Peripheral blood mononuclear cells (PBMCs) were isolated and NK cell (CD3^−^, CD172^−^, CD8^+^) frequencies were determined by flow cytometry at post infection day (PID) 0, 6–8, and 12–17. **(B)** PBMCs and CFSE-stained K562 tumor cells were used as effector and target cells, respectively, and co-cultured at 10:1 5:1 and 1:1 ratios to assess NK cell cytotoxic function at PID 6–8. **(C)** Serum cytokine concentrations (pg/ml) of IL-12 and **(D)** IL-22 were measured at PID 0, 6–8, and 12–17. Asterisks indicate significant differences among treatment groups (mean ± SEM). Statistical analysis was performed using the Student's *t*-test **(B)** or two-way ANOVA with repeated measures and Bonferroni's correction for multiple comparisons **(A,C,D)**. ^*^P < 0.05, ^**^P < 0.01.

Circulating NK cell frequencies were significantly higher in first trimester gilts at PID 0 compared with second or third trimester gilts (Figure 3A). At PID 6-8, peripheral NK cell activity was significantly higher in first compared with second and third trimester gilts (Figure 3B). Mean concentrations of serum IL-12, a cytokine essential for NK cell activation (29) and IL-22, a cytokine produced by subsets of T and NK cells (30–32), were numerically higher at PID 0 in first compared with second and third trimester gilts (Figures 3C,D). It is unlikely changes in serum cytokine concentrations were due to T cells as the frequency of circulating CD4^+^ or CD8^+^ cells did not significantly differ between treatment groups (data not shown). Third trimester gilts had numerically higher mean concentrations of serum proinflammatory cytokines tumor necrosis factor (TNF)-α, interferon (IFN)-α and IL-17 at PID 12–17 compared with first and second trimester gilts (Figures S3A–C). This time point corresponds to gestation day (GD) 104–114 in third trimester gilts, suggesting innate, and proinflammatory cytokines increase immediately prior to parturition in swine, as demonstrated in human pregnancy (33–36). Additionally, third trimester gilts had numerically higher mean serum concentrations of T-helper cell type-1 (Th1) cytokine IFN-γ while first trimester gilts had numerically higher mean serum Th2 and T regulatory cytokines IL-4 and IL-10, respectively, compared with second and third trimester gilts at PID 6–8 (Figures 3SD–F).

### Second Trimester Gilts Had Significantly Higher Circulating PEDV Specific IgA and IgG ASCs, PEDV IgA Abs, and Concentrations of Serum Cytokine Transforming Growth Factor (TGF)-β

**Figure 4:**
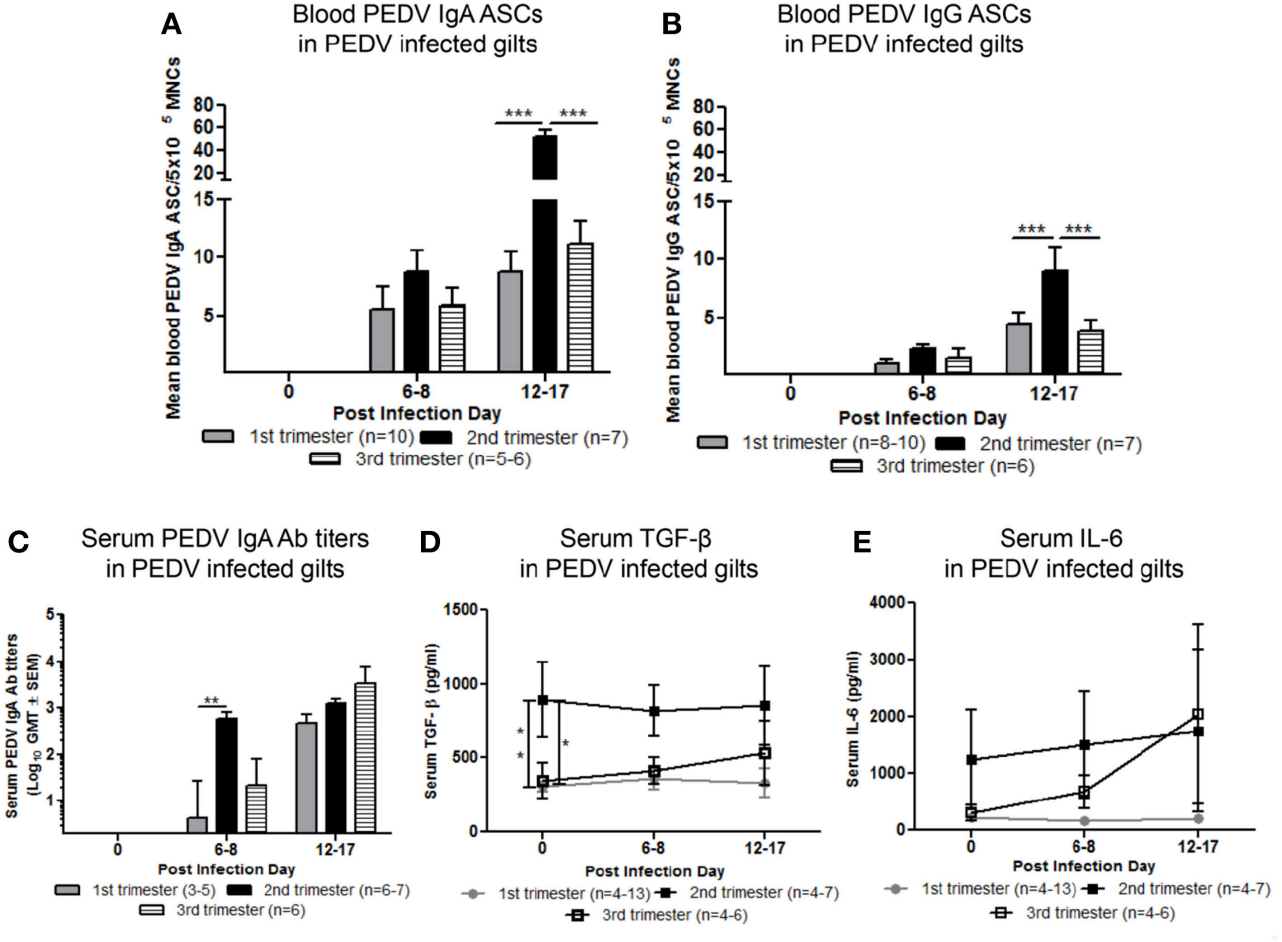
Second trimester gilts had significantly increased circulating PEDV specific IgA and IgG antibody secreting cells (ASCs) at post infection day (PID) 12–17, PEDV specific IgA antibodies (Abs) at PID 6–8 and concentrations of transforming growth factor (TGF)-β at PID 0 compared with first and third trimester gilts. **(A)** Peripheral blood mononuclear cells (PBMCs) were isolated and added to PEDV ELISPOT plates to determine the PEDV specific IgA and **(B)** IgG ASCs. **(C)** Serum PEDV IgA Ab titers and cytokine concentrations (pg/ml) of **(D)** TGF-β and **(E)** interleukin (IL)-6 were determined by ELISA. Gilts were sampled at PID 0, 6–8, and 12–17. Asterisks indicate significant differences among treatment groups at the same time point (mean ± SEM). Statistical analysis was performed using the two-way ANOVA with repeated measures and Bonferroni's correction for multiple comparisons. ^*^P < 0.05, ^**^P < 0.01, ^***^P < 0.001.

Maternal B-cell immune responses were measured at PID 0, 6–8, and 12–17 in first, second and third trimester gilts. Second trimester gilts had significantly higher circulating PEDV IgA and IgG ASCs at PID 12–17 compared with first and third trimester gilts (Figures 4A,B). Additionally, numbers of circulating PEDV IgA ASCs were consistently higher than PEDV IgG ASCs in first, second and third trimester gilts at PID 6–8 and 12–17 (Figures 4A,B). PEDV IgA Ab titers were significantly higher in second trimester gilts at PID 6–8 compared with first trimester gilts (Figure 4C). Serum TGF-β, a cytokine important for IgA class-switching (37), was significantly higher at PID 0 and remained numerically higher at PID 6–8 and 12–17 in second compared with first and third trimester gilts (Figure 4D). Serum IL-6, a cytokine essential for Ab production (38), was numerically higher at PID 0 and 6–8 in second trimester compared with first and third trimester gilts (Figure 4E). No significant differences were observed for serum PEDV IgG or VN Ab titers within the first 2 weeks post-PEDV infection but there was a trend for higher mean titers in second trimester gilts (Figures S4A,B).

### Second Trimester Gilts Maintained Significantly Higher Levels of PEDV Specific ASCs and Abs Throughout Gestation

**Figure 5:**
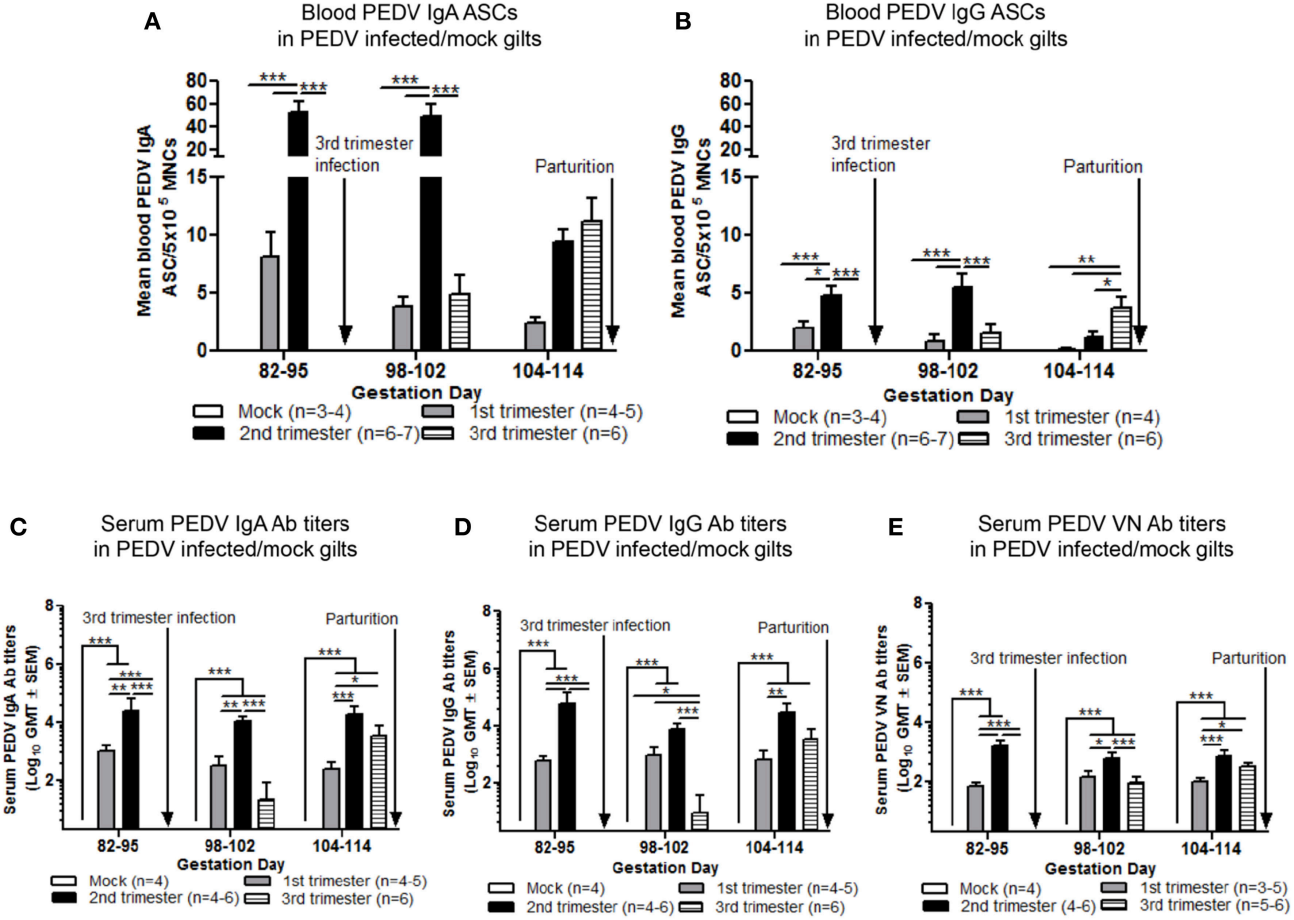
Second trimester gilts maintained elevated circulating PEDV specific IgA and IgG antibody secreting cells (ASCs) and serum PEDV specific IgA, IgG and virus neutralizing (VN) antibodies (Abs) in late pregnancy. **(A)** Peripheral blood mononuclear cells (PBMCs) were isolated and added to PEDV ELISPOT plates to determine the PEDV specific IgA and **(B)** IgG ASCs. **(C)** Serum PEDV IgA and **(D)** IgG Abs were determined by ELISA while **(E)** serum PEDV VN Ab responses were determined by VN Ab assay. Gilts were sampled at gestation day (GD) 82–95, 98–102, and 104–114. Asterisks indicate significant differences among treatment groups at the same time point (mean ± SEM). Statistical analysis was performed using the two-way ANOVA with repeated measures and Bonferroni's correction for multiple comparisons. ^*^P < 0.05, ^**^P < 0.01, ^***^P < 0.001.

To standardize ASC and Ab responses among gilt treatment groups at uniform GDs, circulating PEDV IgA and IgG ASCs, Abs and VN Abs were compared at GD 82–95, 98–102, and 104–114 in first, second, and third trimester PEDV-infected gilts and third trimester mock gilts. Second trimester gilts had significantly higher circulating PEDV IgA and IgG ASCs at GD 82–95 and 98–102 and compared with first and third trimester gilts (Figures 5A,B). PEDV IgA ASCs were consistently higher than PEDV IgG ASCs in blood in first, second and third trimester gilts at GD 82–95, 98–102, and 104–114 (Figures 5A,B). Additionally, circulating PEDV IgA and IgG ASCs in first and second trimester gilts continually decreased in the 3 weeks prior to parturition, demonstrating parturition related or time post-infection effect on circulating PEDV IgA and IgG ASCs. This was not observed in third trimester gilts whose elevated numbers of PEDV IgA (numerically) and IgG (significantly) ASCs at GD 104–114 (Figures 5A,B) corresponded with PEDV RNA shedding in the feces at PID 6–8 and 12–17 (Figure 2A). Second trimester gilts had significantly higher serum PEDV IgA Ab titers compared with first trimester gilts at GD 82–95, 98–102, and 104–114 and third trimester gilts at GD 82–95 and 98–102 (Figure 5C). Serum PEDV IgG Ab titers were significantly higher in second compared with first trimester gilts at GD 82–95 and 104–114 and third trimester gilts at GD 82–95 and 98–102 (Figure 5D). Similarly, PEDV VN Ab titers were significantly higher in second compared with first trimester gilts at all time points but only significantly higher than third trimester gilts at GD 82–95 and 98–102 (Figure 5E).

### Stage of Gestation Modulated Phenotypes of Gut Homing Circulating B Lymphocytes Prepartum

**Figure 6:**
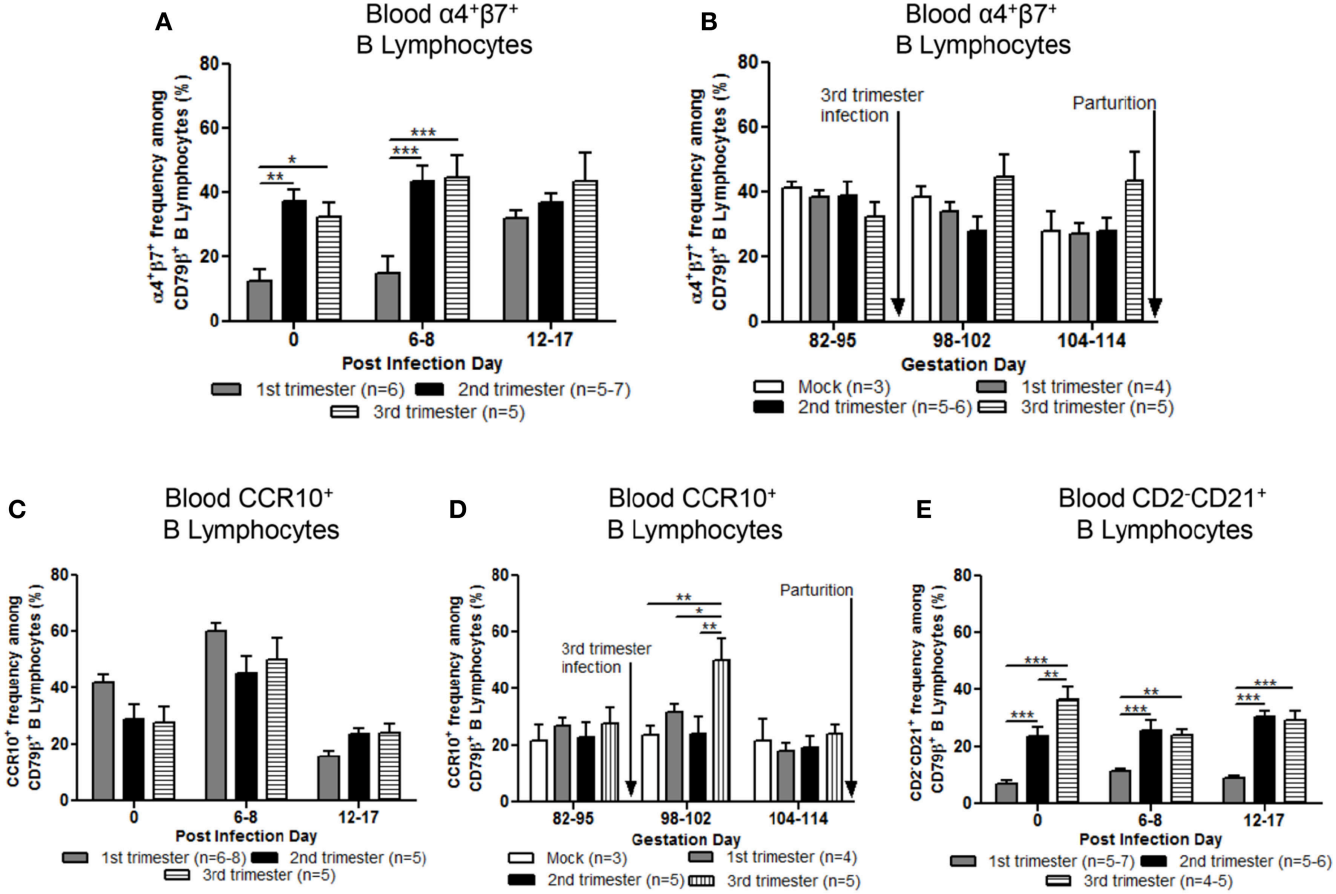
Stage of gestation modulated circulating frequencies of B cell phenotypes prepartum. Peripheral blood mononuclear cells (MNCs) were isolated and analyzed at post infection day (PID) 0, 6–8 and 12–17 and gestation day (GD) 82–95, 98–102, and 104–114 for mean frequencies of α4^+^β7^+^ **(A,B)** and chemokine receptor type 10 (CCR10^+^) **(C,D)** B cells. Additionally, **(E)** primed and/or activated B lymphocytes (CD2^−^CD21^+^) were analyzed at PID 0, 6–8, and 12–17. Asterisks indicate significant differences among treatment groups at the same time point (mean ± SEM). Statistical analysis was performed using the two-way ANOVA with repeated measures and Bonferroni's correction for multiple comparisons. ^*^P < 0.05, ^**^P < 0.01, ^***^P < 0.001.

Mean frequencies of circulating α4^+^β7^+^ (gut homing phenotype) B lymphocytes in second and third trimester gilts were significantly higher compared with first trimester gilts at PID 0 and 6–8 (Figure 6A). Additionally, first trimester gilts had a delayed increase in α4^+^β7^+^ B lymphocyte frequencies in blood post PEDV infection (Figure 6A). Frequencies of α4^+^β7^+^ B lymphocytes were standardized among gilt treatment groups at uniform GDs in late pregnancy (Figure 6B). Third trimester gilts had numerically elevated frequencies of circulating α4^+^β7^+^ B lymphocytes (Figure 6B) at GD 98–102 and 104–114 corresponding with PEDV RNA shedding in the feces at PID 6–8 and 12–17 (Figure 2A). The mean frequencies of circulating chemokine receptor type 10 (CCR10^+^) B lymphocytes were also measured post PEDV infection (Figure 6C) and standardized among gilt treatment groups at uniform GDs in late pregnancy (Figure 6D). No statistical differences among treatment groups were observed at PID 0, 6–8 or 12–17 (Figure 6C). However, third trimester gilts had significantly higher frequencies of circulating CCR10^+^ B lymphocytes at GD 98–102 (PID 6–8) compared with second and first trimester and mock gilts (Figure 6D). The increase in circulating CCR10^+^ B lymphocytes at GD 98–102 corresponded to peak PEDV RNA shedding titers at PID 6–8 (Figure 2A) in third trimester gilts. Additionally, significantly higher frequencies of activated B lymphocytes [CD2^−^CD21^+^ (39)] were observed in the blood of second and third compared with first trimester gilts at PID 0, 6–8, and 12–17 (Figure 6E).

### Second Trimester PEDV-Infected Gilts Provided Optimum Lactogenic Immune Protection Resulting in 100% Piglet Protection

**Figure 7:**
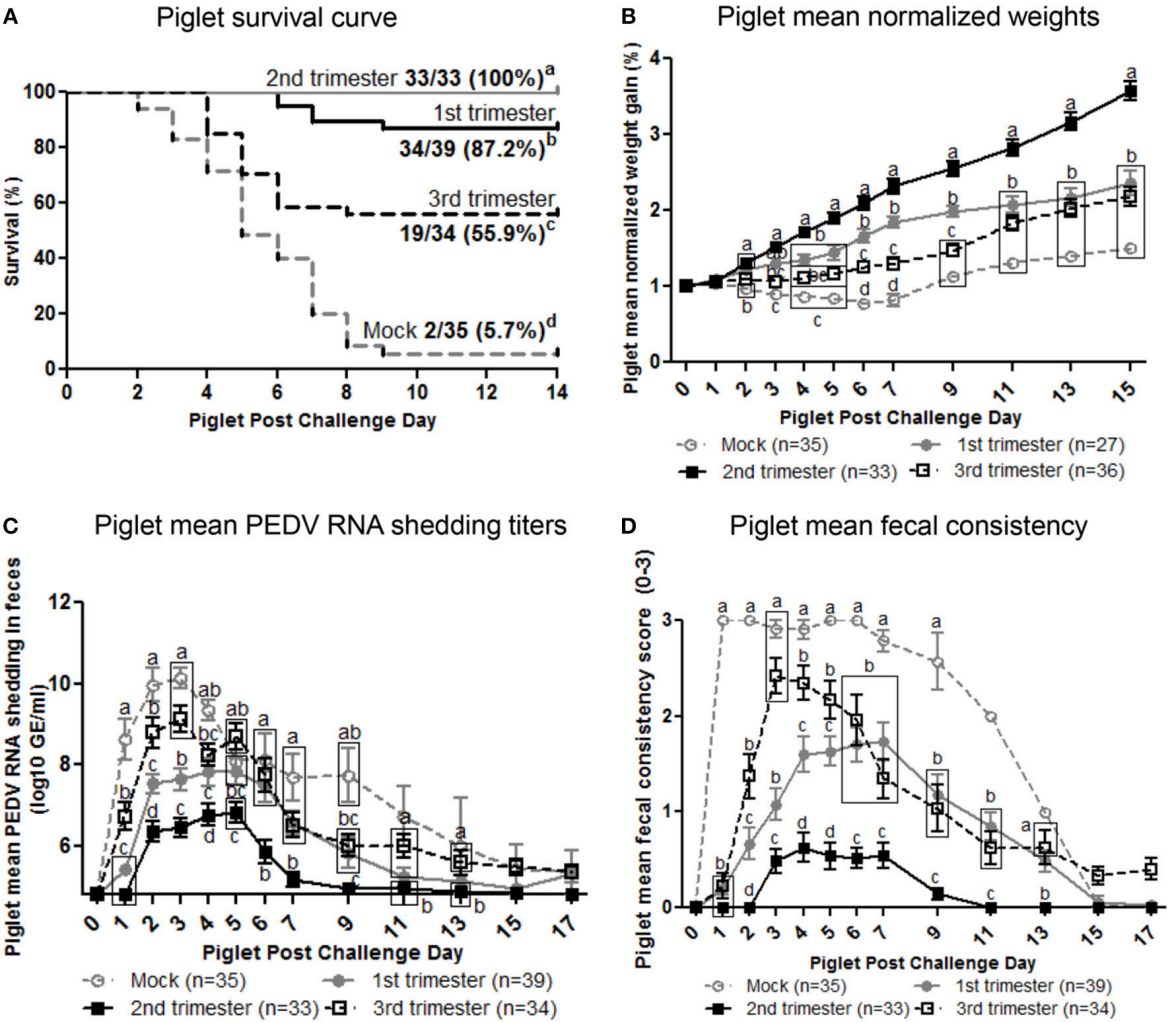
Second trimester PEDV-infected gilts provided optimum lactogenic immune protection compared with first and third trimester and mock gilts. **(A)** Kaplan-Meier survival curve of first, second and third trimester and mock litters at post challenge day (PCD) 0–14. **(B)** Normalized weight gain of first, second and third trimester PEDV-infected and mock litters at PCD 0–15. Weights were normalized by dividing the daily weight (lbs.) by PCD 0 weight (lbs.). **(C)** Piglet PEDV RNA shedding titers were determined by real time quantitative polymerase chain reaction (qRT-PCR) and expressed as log_10_ copies/ml. **(D)** Piglet diarrhea was determined by fecal consistency score >1 (fecal consistency was scored as follows: 0, normal; 1, pasty/semiliquid; 2, liquid; 3, watery). Piglet diarrhea scores and PEDV RNA shedding titers were measured at PCD 1–7, 9, 11, 13, 15, and 17. Different letters indicate significant differences among treatment groups at the same time point (mean ± SEM). Statistical analysis was performed using the two-way ANOVA with repeated measures and Bonferroni's correction for multiple comparisons.

To determine the effect of stage of gestation at time of PEDV infection on lactogenic immune protection, piglets were challenged with PEDV at 3–5 days of age. The number of viable piglets born were not statistically significant between treatment groups (Figure S5). Second trimester PEDV infection of gilts resulted in 100% survival of PEDV challenged piglets (Figure 7A). First trimester gilts provided intermediate protection (87.2% survival) while third trimester gilts provided the least amount of protection (55.9% survival) among PEDV-infected gilts. Mock piglet's survival rate (5.7%) was significantly lower than all other treatment groups. Comparison of piglet weight gain revealed second trimester litters gained significantly more weight than all other treatment groups starting at post challenge day (PCD) 4 and lasting throughout the experiment (Figure 7B). First trimester litters had significantly higher normalized weights than third trimester litters at PCD 6, 7, and 9, but were similar thereafter. Mock litters were stunted (decreased or no weight gain) from PCD 1–7 and had the lowest normalized weights throughout the study. Lactogenic immune protection coincided with decreased PEDV RNA shedding titers where mean peak titers were 6.1 ± 0.2 log_10_ copies/ml, 7.8 ± 0.3 log_10_ copies/ml, 9.1 ± 0.3 log_10_ copies/ml, and 10.1 ±.2 log_10_ copies/ml for second, first, third, and mock litters, respectively (Figure 7C). Corresponding to increased weight gain and decreased PEDV RNA shedding titers, diarrhea scores were significantly lower in second trimester litters throughout the study (Figure 7D). While first trimester litters had diarrhea, it was delayed and significantly lower than third trimester litters at PCD 2–5. Mock litters had diarrhea immediately at PCD 1, lasting through PCD 11 and diarrhea scores were significantly higher than all other treatment groups. Gilt mean PEDV RNA shedding titers and fecal consistency scores were measured post piglet challenge. Mock gilt PEDV RNA shedding titers were significantly higher at PCD 2–7 than first, second and third trimester PEDV-infected gilts and remained numerically higher until PCD 17 (Figure S6A). Additionally, first trimester gilt PEDV RNA shedding titers peaked at PCD 4, earlier compared with second and third trimester gilts (Figure S6A). Mock gilt fecal consistency scores were significantly higher at PCD 5–11 compared with first, second and third trimester PEDV-infected gilts (Figure S6B). Diarrhea was not observed in previously PEDV-infected gilts post piglet challenge (Figure S6B).

### Second Trimester Litters Had the Highest Titers of Circulating PEDV IgA and IgG Abs Positively Correlating With Survival Rate Protection Post PEDV Challenge

**Figure 8:**
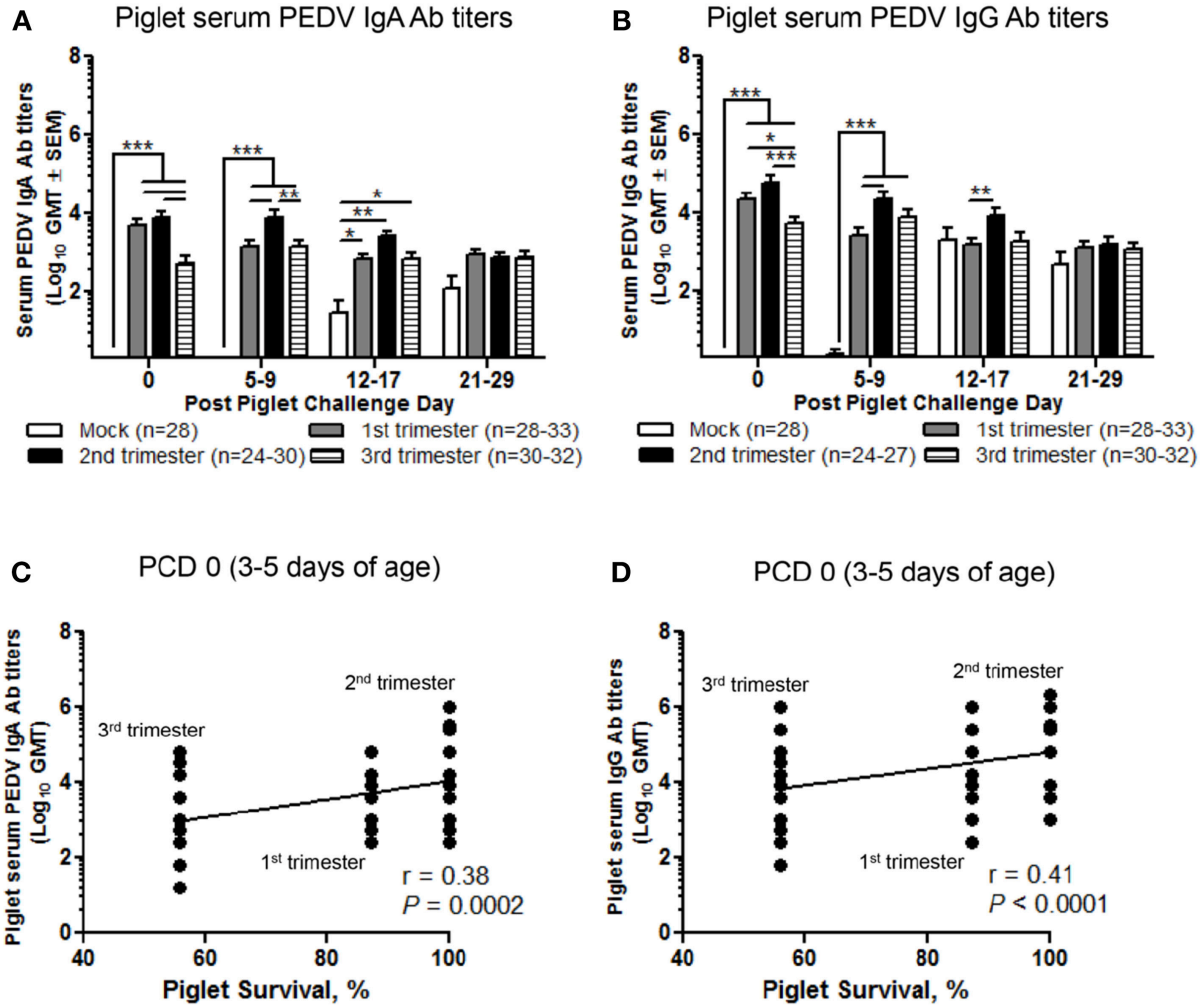
Second trimester litters had the highest peak titers of circulating PEDV IgA and IgG antibodies (Abs) correlating with piglet protection post PEDV challenge. **(A)** Serum PEDV IgA and **(B)** IgG Ab responses were determined by PEDV ELISA assay. Piglet survival rates were significantly positively correlated with circulating PEDV **(C)** IgA and **(D)** IgG Abs at post challenge day (PCD) 0. Piglets were sampled at PCD 0, 5–9, 12–17, and 21–29. Asterisks indicate significant differences among treatment groups at the same time point (mean ± SEM). Statistical analysis was performed using the two-way ANOVA with repeated measures and Bonferroni's correction for multiple comparisons **(A,B)** or Spearman's non-parametric correlation **(C,D)**. ^*^P < 0.05, ^**^P < 0.01, ^***^P < 0.001.

We evaluated the serum titers of piglet PEDV IgA and IgG Abs. Second trimester litters had significantly higher titers of circulating PEDV IgA Abs (Figure 8A) compared with first trimester litters at PCD 5–9 and PEDV IgG Abs at PCD 5–9 and 12–17 (Figure 8B). Additionally, compared with third trimester litters, second trimester litters had higher titers of PEDV IgA Abs at PCD 0 and 5–9 (Figure 8A) and PEDV IgG Abs at PCD 0 (Figure 8B). First trimester litters had significantly higher PEDV IgA and IgG Ab titers at PCD 0 compared with third trimester litters (Figures 8A,B). Lastly, circulating PEDV IgA and IgG Abs at PCD 0 were positively correlated with survival rates post piglet PEDV challenge (Figures 8C,D).

### Piglet Survival Rates Post PEDV Challenge Correlated With Increased PEDV IgA ASCs and Abs and VN Abs in Milk

**Figure 9:**
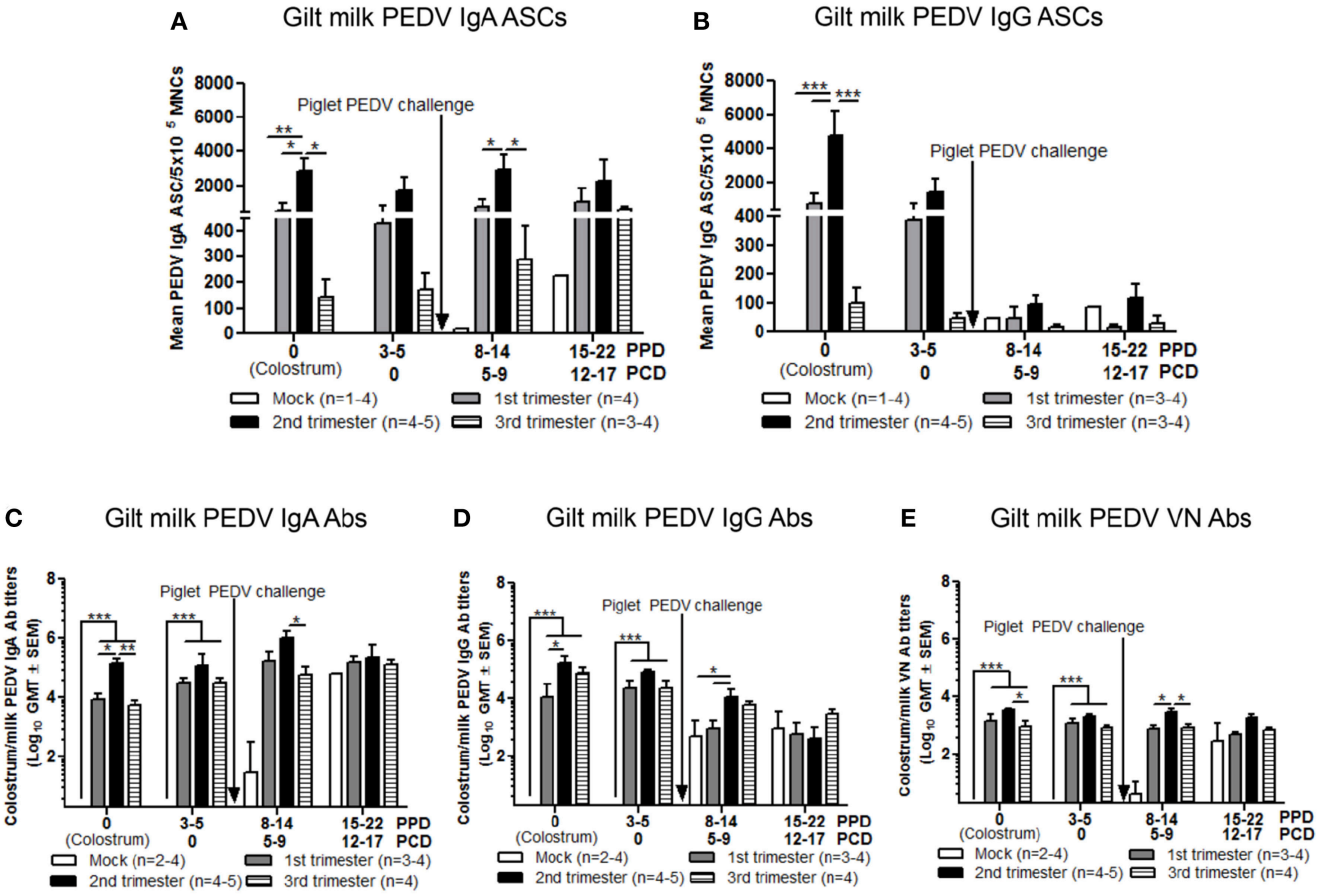
Second trimester gilts had significantly increased circulating PEDV specific IgA and IgG antibody secreting cells (ASCs), PEDV IgA and IgG antibodies (Abs) and PEDV virus neutralizing (VN) Abs in colostrum/milk. **(A)** Milk mononuclear cells (MNCs) were isolated and added to PEDV ELISPOT plates to determine the PEDV specific IgA and **(B)** IgG ASCs. Whey PEDV **(C)** IgA and **(D)** IgG Abs were determined by ELISA while **(E)** whey PEDV VN Ab responses were determined by VN Ab assay. Gilts were sampled at postpartum day (PPD) 0, 3–5 (post challenge day [PCD] 0), 8–14 (PCD 5–9), and 15–22 (PCD 12–17). Asterisks indicate significant differences among treatment groups at the same time point (mean ± SEM). Statistical analysis was performed using the two-way ANOVA with repeated measures and Bonferroni's correction for multiple comparisons. ^*^P < 0.05, ^**^P < 0.01, ^***^P < 0.001.

In colostrum, PEDV IgA and IgG ASCs were significantly higher in second compared with first and third trimester gilts (Figures 9A,B). However, in milk only PEDV IgA ASCs were significantly higher at postpartum day (PPD) 8–14/PCD 5–9 in second trimester gilts compared with all other treatment groups (Figure 9A). While PEDV IgA ASCs were maintained at high numbers in milk throughout lactation, milk PEDV IgG ASCs decreased significantly throughout the study (Figures 9A,B). Similar to ASCs, PEDV IgA and IgG Abs were significantly higher in the colostrum of second compared with first (IgA and IgG) and third trimester (IgA) gilts (Figures 9C,D). Additionally, PEDV VN Ab titers were significantly higher in colostrum in second compared with third trimester gilts (Figure 9E). After PEDV piglet challenge (PPD 8–14/PCD 5–9), PEDV IgA Ab titers in milk were significantly higher in second compared with third trimester gilts while PEDV IgG Ab titers were significantly higher in second compared with first trimester gilts (Figures 9C,D). Lastly, PEDV VN Ab titers were significantly higher in milk at PPD 8–14/PCD 5–9 compared with first and third trimester gilts (Figure 9E). We observed a significant correlation between PEDV IgA and IgG ASCs, and IgA and VN Abs in colostrum and piglet survival (Table 2). However, in mid and late lactation milk, only PEDV IgA ASCs, and IgA and VN Abs were significantly correlated with piglet survival. No significant (*P* < 0.05) correlations were observed between milk IgG ASCs or Abs and piglet survival (Table 2). These correlations are consistent with our hypothesis that IgA ASC and Ab titers in milk are responsible for lactogenic immune protection in neonatal piglets against PEDV challenge.

### PEDV Antibody^+^ Cells Increased in the MG Post PEDV Challenge

Third trimester gilts had lower mean numbers of PEDV Ab^+^ cells in the MG (2.7 ± 0.7) per microscopic field (30×) compared with second (6.1 ± 3.2) and first (6.9 ± 1.4) trimester gilts at GD 104–114 (Figure S7). There were no PEDV Ab^+^ cells in the MG of mock gilts prepartum (GD 104–114). This suggests that while not significantly different, the mean numbers of PEDV Ab^+^ cells in the MG may be reflective of levels of PEDV ASCs and Abs in colostrum. For example, third trimester gilts had the lowest mean number of PEDV Ab^+^ cells per microscopic field in the MG prepartum coinciding with the lowest mean numbers of IgA and IgG ASCs and mean titers of PEDV IgA and VN Abs in colostrum. PEDV Ab^+^ cells were also observed in the lactating MG at PCD 5–9. First, second and third trimester PEDV-infected gilts had significantly higher numbers of PEDV Ab^+^ cells per microscopic field in the MG compared with mock gilts post piglet challenge (Figure S7).

### PEDV Exposure Post Piglet Challenge Differentially Affects MG, Spleen, Mesenteric Lymph Node, and Ileum ASC Responses of Gilts

The highest mean numbers of PEDV IgA ASCs were in the ileum while the highest mean numbers of PEDV IgG ASCs were in spleen and ileum (Figures S8A,B). Second trimester gilt mean numbers of IgA and IgG ASCs were significantly higher in the ileum than third and first trimester gilts, respectively. However, mock gilts had similar or higher mean numbers of IgA and IgG ASC in the spleen, mesenteric lymph node (MLN), and ileum compared with first, second, and third trimester gilts (Figures S8A,B), corresponding to higher PEDV RNA shedding titers in feces and PEDV-induced diarrhea of mock gilts (Figures S6A,B). Due to the high mortality rate of mock litters (Figure 7A), the MGs of mock gilts regressed rapidly post piglet challenge (40). There was not enough MG tissue left at PCD 21–29 to collect MG MNCs for ASC analysis.

## Discussion

Diarrheal disease represents a major global health burden and is the leading cause of morbidity and mortality in young children and animals. Specifically, the emergence of PEDV in the US in 2013 led to over 8.5 million piglet deaths and an estimated multi-million-dollar loss to the US swine industry (3, 41). Maternal vaccination that enhances passively transferred protective Abs in milk, induced via the gut-MG-sIgA axis, is the major strategy to protect neonatal suckling piglets immediately after birth (4, 5). However, due to the biological and immunological differences at each stage of pregnancy (18), the optimum time to induce maternal immunity and the gut-MG-sIgA axis was previously undefined. Here, we evaluated the impact of stage of gestation at PEDV infection on the maternal immune response, the generation of PEDV-specific ASCs and Abs in serum and milk and the protective effects of lactogenic immunity on PEDV-challenged piglets.

A major finding of our study is that PEDV infection in the second trimester was the optimum stage of gestation to generate the highest maternal immune responses in blood and milk correlating to 100% lactogenic immune protection in PEDV challenged suckling piglets (Table 1). For example, second trimester gilts had the highest peak levels of circulating PEDV IgA and IgG ASCs and Abs and VN Abs prior to parturition. Interestingly, this coincided with higher serum cytokine concentrations of TGF-β (significantly) and IL-6 (numerically) at PID 0 in second trimester gilts. TGF-β a cytokine produced by multiple lineages of leukocytes and stromal cells (42), is required for IgA class switch recombination (43) and IgA^+^ B cell survival in the lamina propria (44). Additionally, IL-6 promotes Ab production by increasing B cell helper capacity of CD4^+^ T cells (38). Our data support previous research demonstrating increased serum TGF-β in women during the second trimester (45) correlating with increased Ab titers (46). The increased serum TGF-β and IL-6 at PEDV infection in second trimester gilts likely contributed to the enhanced anti-PEDV maternal immune responses.

Despite being PEDV-infected 5 weeks earlier, first trimester gilts generated a lower maternal immune response during gestation compared with second trimester gilts (Table 1). This suggests stage of gestation dependent differences in immune parameters may contribute to differential induction of the humoral immune response during PEDV infection (47, 48). The increase in NK cell frequencies at PID 0 in first compared with second and third trimester gilts (first trimester, GD 19–22; second trimester, GD 57–59; and third trimester, GD 96–97) is in agreement with previous studies reporting increased numbers of cytotoxic peripheral NK cells in first trimester women and swine (49–51). Furthermore, the significantly higher circulating NK cell cytotoxicity at PID 6–8 coincided with enhanced mean concentrations of serum IL-12 and IL-22, NK cell activation, and proliferation cytokines, at PID 0 in first trimester gilts (29, 52–54). Increased peripheral and decidual NK cell frequencies and lytic activity during the first trimester are thought to support maternal tissue remodeling during embryo implantation in swine and humans (20, 51, 55, 56). Additionally, NK cells are known to modulate B cell immunity (57, 58). Therefore, the unique immune environment during the first trimester may impact B cell responses to PEDV infection in pregnant gilts. Future studies investigating the effects of NK cells on the maternal immune response during an enteric viral infection in gilts are warranted.

We also hypothesized that stage of gestation would impact B cell phenotypes pre and post PEDV infection. For example, lymphocyte α4^+^β7^+^ integrin is a mucosal adhesion molecule responsible for cellular migration in the intestinal mucosa and lymphoid tissue by interacting with its receptor mucosal addressin cellular adhesion molecule 1 (MAdCAM-1) (59–62). Circulating α4β7^+^ lymphocytes reflect the intestinal tissue in which they were primed (63) and may influence the gut-MG-sIgA axis. The significantly higher frequencies of α4^+^β7^+^ B cells at PID 0 and 6–8 in second and third trimester, compared with first trimester gilts (Table 1), coincides with increased concentrations of immune modulating pregnancy-associated hormones in swine, like estrone, estrone sulfate, 17β-estradiol (E2), and E2 sulfate (64). For example, trafficking of IgA^+^ lymphoblasts to the MG in ovariectomized female mice was enhanced after E2 administration (65). Additionally, E2 in combination with progesterone administration in ovariectomized female mice resulted in increased L-selectin and α4-dependent adhesion of NK cells to mucosal tissues (60). Additionally, we observed differential effects of gestational stage on CD2^−^CD21^+^ (primed and/or activated) B cell frequencies in blood. The significant increase in circulating CD2^−^CD21^+^ B cell frequencies at PID 6–8 and 12–17 in second and third trimester compared with first trimester gilts is similar to the trend observed for α4β7^+^ B cell frequencies later in pregnancy (Table 1). The increasing concentrations of pregnancy-associated hormones as pregnancy progresses could modulate circulating frequencies of CD2^−^CD21^+^ B cells, contributing to the differential immune responses observed in this study. Future studies treating ovariectomized swine with exogenous E2 or progesterone are needed to decipher the *in vivo* effects of pregnancy associated hormones on mucosal lymphocyte trafficking, B cell activation and ASC and Ab production.

To our knowledge, this is the first study to demonstrate an effect of stage of gestation at time of enteric viral infection on the gut-MG-sIgA axis and immune protection in suckling piglets. In our study, second trimester gilts provided greater passive lactogenic immune protection (100%) compared with first (87.2%) and third (55.9%) trimester and mock gilts (5.7%). The increased levels of protection were significantly correlated with PEDV IgA ASCs and Abs and VN Abs in colostrum and milk (Tables 1, 2). These results are in agreement with previous work done with TGEV-infected pregnant swine. For example, high rates of protection against TGEV in piglets were achieved when pregnant sows were orally infected (6, 7, 13) with live virulent virus (14, 15). The increased rate of protection was associated with high titers of IgA Abs in colostrum and milk induced via the gut-MG-sIgA axis (66). Additionally, PEDV IgA and IgG Ab titers in piglet serum at PCD 0 correlated with piglet protection against PEDV challenge. In our study, we experimentally infected gilts with the same dose and strain of PEDV and controlled for parity and lack of prior TGEV-exposure. Therefore, we were able to observe correlates of immunity where second trimester gilts provided optimal protection in PEDV challenged suckling piglets correlating with levels of colostrum and milk PEDV IgA ASCs and Abs and VN Abs and piglet serum IgA and IgG Abs (Table 1). Experimental infection studies of gilts/sows with controlled PEDV doses and timing of piglet challenge post parturition are important for determining correlates of lactogenic immune protection against PEDV.

Lastly, we observed significantly higher PEDV RNA shedding titers at PID 2 and diarrhea scores at PID 4 in third trimester gilts. This suggests factors involved in intestinal homeostasis are impacted by the hormonal changes during the third trimester. For example, circulating E2 and porcine growth hormone concentrations increase in the third trimester prior to parturition (64). There is growing evidence that hormones dynamically influence the gut microbiome (67). Coincidently, the gut microbial environment during pregnancy changes dramatically from the first to third trimesters in humans (68, 69) and swine (70) resulting in decreased bacterial community richness. Additionally, studies of PEDV-infected pregnant sows revealed a significant decrease of observed bacterial species compared with healthy pregnant sows (71). Although the gut microbiome was not examined in this study, it is possible that pregnancy hormone-induced effects on the gut microbiome during late pregnancy in combination with PEDV infection resulted in increased gut dysbiosis in third trimester pregnant gilts. Future studies investigating the impact of the microbiome during pregnancy on PEDV pathogenesis and the gut-MG-sIgA axis are warranted.

Previous research investigating stage of gestation at vaccination on the immune response in pregnant women reached conclusions similar to ours. Women vaccinated with the tetanus-diphtheria-acellular pertussis (Tdap) vaccine in the second and/or early third trimester had a higher concentration and avidity of anti-Tdap Abs in maternal blood and increased transplacental anti-Tdap Ab transfer compared with women immunized in the mid to late third trimester (72–74). This is in agreement with our results, demonstrating that second trimester PEDV infection yields the highest titer of PEDV IgA, IgG, and VN Abs in serum and colostrum/milk. More research is needed in humans and livestock to better understand maternal immunity and to design the most effective maternal vaccines to passively protect suckling neonates and prevent neonatal morbidity and mortality.

In summary, a comparison of PEDV infection at different stages of gestation demonstrated that first, second and third trimester gilt immune responses vary greatly in magnitude of their immune response and association with lactogenic immune protection. We conclude that second trimester gilts have the greatest capacity to generate a robust anti-PEDV humoral immune response prior to parturition, resulting in the highest amount of lactogenic immune protection against PEDV challenge in neonatal suckling piglets. Additionally, piglet protection against PEDV challenge is correlated with PEDV IgA ASC, IgA Abs, and VN Abs in milk and PEDV IgA and IgG Abs in piglet serum (Tables 1, 2). Our results provide novel insights and identify possible predictors of maternal vaccine efficacy for passive protection of suckling piglets. Finally, pregnancy should not be evaluated as a single event and development of a successful attenuated PEDV vaccine requires consideration of stage of gestation at the time of vaccination.

## Materials and Methods

### Virus

The wild-type PC22A strain of PEDV was used for gilt infection and piglet challenge at a dose of 1 × 10^5^ plaque forming units (PFU) diluted in Minimal Essential Media [MEM (Life Technologies, Carlsbad, CA)]. Briefly, PC22A was isolated and cultured in Vero cells as described previously (75, 76). Cells were grown in growth medium containing Dulbecco's Modified Eagle's Medium [DMEM (Life Technologies, Carlsbad, CA)] supplemented with 5% fetal bovine serum (Life Technologies, Carlsbad, CA) and 1% antibiotic-antimycotic (Life Technologies, Carlsbad, CA). Virus was grown in Vero cells in maintenance medium containing DMEM supplemented with 10 μg/ml trypsin (Life Technologies, Carlsbad, CA), 0.3% tryptose phosphate broth (Sigma Aldrich, St. Louis, MO), and 1% antibiotic-antimycotic. Cells were kept in a humidified incubator at 37°C and 5% CO_2_. PC22A was passaged three times in Vero cells before passaging once for generation of inoculum in a gnotobiotic pig. The virulence of pig passaged PC22A was confirmed in adult and neonatal pigs (4, 77, 78). Cell-culture adapted PC22A was used as a positive control in the VN Ab assay.

### Experimental Design

All animal experiments were approved by the Institutional Animal Care and Use Committee at The Ohio State University. All pigs were maintained, sampled, and euthanized humanely. First parity PEDV, porcine deltacoronavirus (PDCoV) and TGEV seronegative pregnant gilts (Landrace × Yorkshire × Duroc cross-bred) were acquired from either Wilson Farms (Elkhorn, WI) or The Ohio State University swine center facility and randomly assigned to one of four treatment groups: PEDV-infected (1) first trimester (GD 19–22) (*n* = 10); (2) second trimester (GD 57–59) (*n* = 7); or (3) third trimester (GD 96–97) (*n* = 6) or MEM-infected third trimester control [GD 96–97 (*n* = 4)] (Figure 1A). Second and third trimester gilts arrived at our facilities at GD 49–51 and 89–92, respectively, and acclimated for 1 week prior to PEDV infection. First trimester gilts were housed and PEDV-infected at the National Animal Disease Center, USDA-ARS, in Ames, Iowa and then four gilts were shipped to our facilities at GD 93 after they were determined to be fecal shedding negative by real-time quantitative polymerase chain reaction (RT-qPCR). Gilt fecal samples were collected, and clinical signs observed on PID 0, 2, 4, 6, 10, and 14. Fecal consistency was scored as follows: 0, solid; 1, pasty; 2, semi-liquid; 3, liquid, respectively. A fecal consistency score of >1 was considered as diarrhea (4, 77). Blood samples were collected post-PEDV infection at PID 0, 6–8 and 12–17 and also prior to parturition at GD 82–95, 98–102, and 104–114 for serum and mononuclear cell (MNC) isolation. All gilts farrowed naturally in our facilities at GD 114 (± 3) and colostrum was collected within 12 h of parturition. Piglets were orally PEDV-challenged at 3–5 days of age (mean ± SD challenge day for first trimester litters = 4.0 ± 0.82, second trimester litters = 4.4 ± 0.96, third trimester litters = 4.2 ± 0.82 and mock litters = 3.7 ± 0.96). Gilt and piglet serum were collected on PCD 0, 5–9, 12–17, and 21–29. All colostrum and milk samples were collected after administration of 2cc oxytocin intramuscularly (IM) at PPD 0, 3–5, 8–14, and 15–22 (Figure 1B). Gilt MG biopsies were collected at GD 104–114 and PPD 8–14. Piglet fecal samples were collected and clinical signs and body weights were recorded daily on PCD 0–7 and every other day through PCD 15–17. All animals were euthanized at PCD 21–29. Upon euthanasia, gilt blood, ileum, MG, MLN, and spleen tissues were collected for MNC isolation. Piglet blood was collected and the serum separated for immunologic assays.

### PEDV RNA Quantification by RT-qPCR

To determine PEDV RNA shedding titers, two rectal swabs were suspended in 4 mL MEM as described previously (78). Viral RNA was extracted from 50 μl of fecal supernatants following centrifugation (2,000 × g for 30 min at 4°C) using the MagMAX Viral RNA Isolation Kit (Applied Biosystems, Foster City, CA) according to the manufacturer's instructions. Titers of viral RNA shed in feces were determined by TaqMan RT-qPCR using the Onestep RT-PCR Kit (QIAGEN, Valencia, CA) (78). The detection limit was 10 copies per 20 μL of reaction, corresponding to 4.8 log_10_ copies/mL of original fecal samples.

### Isolation of MNCs in Blood, Spleen, MLN, and Ileum

Blood, spleen, MLN, and ileum were collected aseptically at euthanasia and processed for MNC isolation as described previously (79). The isolated cells were resuspended in enriched RPMI [E-RPMI (Roswell Park Memorial Institute)] medium containing 8% fetal bovine serum, 2 mM l-glutamine, 1 mM sodium pyruvate, 0.1 mM non-essential amino acids, 20 mM HEPES (*N*-2-hydroxy- ethylpiperazine-*N*−2-ethanesulfonic acid), and 1% antibiotic-antimycotic (Life Technologies, Carlsbad, CA) and used for assays. The viability of MNCs was determined by trypan blue exclusion. Briefly, MNCs were diluted two-fold in 0.4% trypan blue before visualizing using an automated cell counter (Cellometer, Nexcelom, Lawrence, MA). The viability (%) was calculated as [1.00–(number of blue cells/numbers of total cells)] × 100.

### Isolation of MNCs From the MG Tissue

The MG was collected aseptically at euthanasia and placed in ice-cold wash medium (RPMI 1640 with 10 mM HEPES, 200 g of gentamicin per ml, and 20 g of ampicillin per ml [Life Technologies, Carlsbad, CA]). Tissue was minced and pressed through stainless steel 80-mesh screens of a cell collector (Cellecter; E-C Apparatus Corp., St. Petersburg, FL.) to obtain single-cell suspensions and then pooled. A 90% Percoll solution (Sigma Aldrich, St. Louis, MO) was added to the MG cell suspensions and centrifuged at 1,200 × g for 30 min at 4°C. Cell pellets were resuspended in 43% Percoll, underlaid with 70% Percoll, and centrifuged at 1,200 × g for 30 min at 4°C. The MNC were collected from the 43-to-70% interface and washed with wash medium. Cells were filtered through a 70 μM pore filter and resuspended in E-RPMI. The viability of MNCs was determined by trypan blue exclusion.

### Detection of Cytokines in Serum by ELISA

Serum samples were processed and analyzed for proinflammatory (TNF-α, IL-6, IL-17, IL-22), innate (IFN-α) and Th1 (IL-12, IFN-γ), Th2 (IL-4), and T regulatory (IL-10 and TGF-β) cytokines as described previously with some modifications (80, 81). Briefly, Nunc Maxisorp 96-well plates were coated with anti-porcine IL-4 (2 μg/ml, clone A155B16F2), anti-porcine IL-10 (4 μg/ml, clone 945A4C437B1), anti-porcine IFN-γ (1.5 μg/ml, clone A151D5B8), anti-porcine TGF-β (1.5 μg/ml, clone 55B16F2) (Thermo Fisher Scientific, Waltham, MA), anti-porcine IL-6 (0.75 μg/ml, goat polyclonal Ab), anti-porcine IL-12 (0.75 μg/ml, goat polyclonal Ab), anti-porcine IFN-α (2.5 μg/ml, clone K9) (R&D systems, Minneapolis, MN), anti-porcine TNF-α (1.5 μg/ml, goat polyclonal Ab), anti-porcine IL-17 (1.5 μg/ml, rabbit polyclonal Ab), and anti-porcine IL-22 (1.5 μg/ml, rabbit polyclonal Ab) (Kingfisher Biotech, Saint Paul, MN) overnight at 37°C for IFN-α or 4°C for all other cytokines. Biotinylated anti-porcine IL-4 (0.5 μg/ml, clone A155B15C6), anti-porcine IL-10 (1 μg/ml, clone 945A1A926C2), anti-porcine IFN-γ (0.5 μg/ml, clone A151D13C5), anti-TGF-β (0.4 μg/ml, TGF-β-1 Multispecies Ab Pair CHC1683) (Thermo Fisher Scientific, Waltham, MA), anti-porcine IL-6 (0.1 μg/ml, goat polyclonal IgG), anti-porcine IL-12 (0.2 μg/ml, goat polyclonal IgG), anti-porcine IFN-α (3.75 μg/ml, clone F17) (R&D systems, Minneapolis, MN), anti-porcine TNF-α (0.4 μg/ml, goat polyclonal Ab), anti-porcine IL-17 (1 μg/ml, rabbit polyclonal Ab) or anti-porcine IL-22 (1 μg/ml, rabbit polyclonal Ab) (Kingfisher Biotech, Saint Paul, MN) were used for detection. Porcine IFN-α detection Ab was biotinylated using a commercial kit as described previously (81). Plates were developed and cytokine concentrations were calculated as described previously (80). Sensitivities for these cytokine ELISA assays were 1 pg/ml for IL-4, IL-12, and IFN-α, 4 pg/ml for TNF-α, IL-17, and IL-22, 8 pg/ml for TGF-β, and 16 pg/ml for IL-6, IL-10, and IFN-γ.

### Colostrum/Milk Processing for Whey and Isolation of MNCs

Colostrum/milk was collected aseptically after gilts were given 2cc oxytocin (VetOne, Boise, ID) IM to facilitate collection of mammary secretions. Colostrum/milk was placed immediately on ice. Samples were filtered through a 70 μM pore filter and centrifuged at 1,800 × g for 30 min at 4°C to separate fat, skim milk, and cell pellet portions. Fat was removed utilizing sterile plain-tipped applicators (Fisher Scientific, Hampton, NH). Skim milk was collected, centrifuged at 28,000 × g for 1 h at 4°C to separate the whey that was then stored at −20°C until tested. The cell pellet portion was resuspended in a 90% Percoll solution and centrifuged at 1,200 × g for 30 min at 4°C. Cell pellets were resuspended in 43% Percoll, underlaid with 70% Percoll, and centrifuged at 1,200 × g for 30 min at 4°C. The MNC were collected from the 43-to-70% interface and washed with wash medium. Cells were filtered through a 70 μM pore filter and resuspended in E-RPMI. The viability of the MNC preparation was determined by trypan blue exclusion.

### NK Cell Cytotoxicity Assay

Blood MNC and K562 cells were used as effector and target cells, respectively. Effector: target cell ratios of 10:1, 5:1, and 1:1 were used and the assay was done as described previously (82).

### Flow Cytometry to Assess NK Cell Frequencies

To determine the frequencies of NK cells, CD3^−^, CD172^−^, CD8^+^ MNCs were identified. Briefly, 100 μl of peripheral blood mononuclear cells (PBMCs) at 1 × 10^7^ cells/ml were stained with anti-porcine CD3 (clone PPT3, Southern Biotech), anti-porcine SWC3a [CD172 human analog (clone 74-22-15, Southern Biotech)] and anti-porcine CD8 (clone 76-2-11, Southern Biotech) monoclonal Abs (mAbs) for 15 min at 4°C. Subsequently cells were washed and incubated with streptavidin APC (BD Biosciences, San Jose, CA, USA) secondary Ab. Appropriate isotype matched control Abs were included. Acquisition of 50,000 events and analyses were done using the Accuri C6 flow cytometer (BD Biosciences). The gating strategy is depicted in Figure S1.

### PEDV Plaque Reduction VN Assay

A plaque reduction VN assay was performed as described previously (83) with modifications. Serum and whey were inactivated at 56°C for 30 min prior to testing for PEDV neutralizing Abs. Serial 4-fold dilutions of test sera or whey were mixed with 70 PFU and 140 PFU PEDV PC22A cell-culture adapted strain, respectively. The serum/whey-virus mixtures (290 μl/well) were incubated for 1.5 h at 37°C with gentle rocking and then duplicate samples were infected onto 2–3 day confluent Vero cell monolayers in 6-well plates. Plates were incubated at 56°C for 1 h, lightly rocking every 15 min. Subsequently, the inoculum was removed, cells were washed twice with sterile PBS [1X pH 7.4 (Sigma Aldrich, St. Louis, MO)] and overlaid with 0.75% low melting point agarose (SeaPlaque, Lonza, Riverside, PA) in serum free media supplemented with tryptose phosphate broth and trypsin as described for PEDV cultivation (75). Plates were incubated at 37°C for 3 days and then stained with 0.001% neutral red solution (Sigma Aldrich, St. Louis, MO). Plaques were counted and the VN titers were determined by calculating the reciprocal of the highest dilution of a serum/whey sample showing an 80% reduction in the number of plaques compared with seronegative control serum/whey.

### PEDV Whole Virus Ab ELISA

Cell culture adapted PC22A was propagated on 2–3-day-old Vero cells in polystyrene roller bottles (Fischer Scientific, Hampton, NH) until the cells demonstrated 90–95% cytopathic effects (CPE). Roller bottles were subjected to two freeze-thaw cycles at −80°C before collecting the supernatant and centrifuging at 3,000 × g for 10 min at 4°C to remove Vero cell debris. The supernatant was overlayed onto 3 ml sucrose [35% in TNC buffer (50 mM Tris at pH 7.4, 150 mM NaCl, 10 mM CaCl_2_, 0.02% NaN_3_)] and centrifuged at 112,700 × g for 2 h at 4°C. The viral pellet was resuspended in TNC buffer, centrifuged at 107,200 × g for 2 h at 4°C and washed twice with sterile PBS (1X pH 7.4). Then the viral pellet was inactivated by using binary ethylenimine (BEI) as previously described (84). The semipurified inactivated virus was resuspended in PBS (1X pH 7.4) at a dilution of 1:3 of the original supernatant volume and stored at −80°C. At the time of testing, PEDV or mock antigen solution was further diluted 1:4 and added (60 μl per well) to uncoated polystyrene plates (Fisher Scientific, Hampton, NH) in alternating wells. Plates were incubated for 4 h at 37°C or overnight at 4°C. Plates were washed 5X with PBS-T wash solution (PBS 1X, 0.1% Tween-20 [Fisher Scientific, Hampton NH], pH 7.4). General Block ELISA blocking solution (Immunochemistry Technologies, Bloomington, MN) was added (200 μl per well) and plates were stored at 4°C overnight. After incubation, plates were washed twice with PBS-T and samples were added. All samples were serially diluted starting at 1:4 and added (50 μl per well) in duplicate to coated plates. Positive and negative controls (Ab-positive and -negative experimental samples) were included per plate for each sample type. Plates were incubated at room temperature (RT) for 1.5 h and washed 5X with PBS-T. Primary Abs were added (100 μl per well) diluted in PBS-T to detect IgA Ab [peroxidase-conjugated goat anti-pig IgA (Bio-Rad, Hercules, CA)] and IgG [biotin-conjugated goat anti-pig IgG (Seracare, Milford, MA)] in serum (1:4,000 and 1:20,000, respectively) and milk/intestinal samples (1:20,000). IgA plates were incubated at RT for 1.5 h and IgG at 37°C for 1 hr. For IgG plates, peroxidase-conjugated streptavidin [1:10,000 (Roche, Basel, Switzerland)] was added (100 μl per well) and incubated at RT for 1 h. For both IgA and IgG, plates were washed twice with PBS-T and the reaction was visualized for all plates by adding 100 μl of 3,3′,5,5′-tetramethylbenzidine (TMB) substrate with H_2_O_2_ membrane peroxidase substrate system (Seracare, Milford, MA). Plates were incubated at RT for 5 min and stopped by addition of 100 μl stop solution (1 M sulfuric acid) to each well. Reactions were measured as optical density at 450 nm using an ELISA plate reader (Spectramax 340pc, Molecular Devices, San Jose, CA). The ELISA Ab titer was expressed as the reciprocal of the highest dilution that had a corrected *A*_450_ value (sample absorbance in the virus-coated well minus sample absorbance in the mock antigen-coated well) greater than the cut-off value (mean corrected *A*_450_ value of negative controls plus 3 standard deviations). Samples negative at a dilution of 1:4 were assigned a titer of 1:2 for the calculation of geometric mean titers (GMTs).

### PEDV Antibody Secreting Cell ELISPOT

To detect PEDV ASCs, PEDV PC22A, and mock-infected, acetone-fixed Vero cells in 96-well plates were used similar to methods used to detect TGEV ASC (85, 86). Briefly, after infected Vero cells (at 95–100% confluency) showed 90–95% CPE, cells were fixed with 80% acetone for 15 min, allowed to dry for 1–2 h and stored at −20°C. Mock-infected cell monolayers served as negative controls. Plates were thawed and rehydrated in sterile PBS for 15 min at RT. Serial dilutions of MNCs (5 × 10^5^, 5 × 10^4^, 5 × 10^3^, and 5 × 10^2^) were added to duplicate wells (100 μl per well) of fixed PEDV PC22A and mock-infected cell monolayers. Plates were centrifuged at 120 × g, for 5 min at RT and incubated at 37°C with 5% CO_2_ for 12–14 h. After incubation, the plates were washed 5X with PBS-T. ASCs were detected by incubating plates with HRP-conjugated anti-pig IgA [1:4,000 (Bio-Rad, Hercules)] or biotinylated anti-pig IgG [1:20,000 (Seracare, Milford, MA)]. All Abs were added at 100 μl per well. Plates incubated with Abs to IgA were incubated at RT for 1.5 h and for Abs to IgG at 37°C for 1 h. For IgG plates, peroxidase-conjugated streptavidin [1:10,000 (Roche, Basel, Switzerland)] was added (100 μl per well) and incubated at RT for 1 h. The reaction was visualized for all plates by adding 100 μl TMB substrate with H_2_O_2_ membrane peroxidase substrate system (Seracare, Milford, MA). Spots were detected and counted using a light microscope. Counts were averaged from duplicate wells and expressed relative to 5 × 10^5^ MNC.

### Histopathologic Analysis and Evaluation of PEDV Ab^+^ Cells in the MG

To evaluate PEDV Ab^+^ cells in the MG, 5 cm biopsies were collected using a 12 gauge × 10 cm semi-automatic needle (Bard Peripheral Vascular, Tempe, AZ) and fixed in 10% neutral buffered formalin. Sections were trimmed, processed, and embedded in paraffin. Sections were cut (3.5 μm thick) and processed for antigen retrieval (0.05% pronase E treatment [Sigma Aldrich, St. Louis, MO]). For detection of PEDV Ab^+^ cells, a PEDV viral suspension sandwich immunohistochemistry (IHC) method was developed. Semipurified inactivated PEDV antigen, as described previously for PEDV Ab ELISA plates, was added to tissue sections and incubated overnight at 4°C. A mAb to the N protein of a highly virulent US PEDV strain [PC22A-like (PEDV 72-111-25 IgM G-14, kindly provided by Steven Lawson and Eric Nelson, Department of Veterinary and Biomedical Sciences, South Dakota State University)] was added to tissue sections and incubated overnight at 4°C. Supersensitive Polymer-HRP IHC Detection System (Biogenex, Fremont, CA) was used as a secondary Ab and substrate prior to hematoxylin and eosin (H&E) staining. Microscopic imagines (30 × magnification) were obtained using a fluorescence microscope (Olympus IX70-S1F2). Mean numbers of PEDV Ab^+^ cells were evaluated by measuring at least 3 different microscopic fields at (30 × magnification) for each sample time point (GD 104–114 and PCD 5–9) from first, second and third PEDV-infected or mock gilts.

### Flow Cytometry to Assess Lymphocytes and Homing Marker Integrin and Receptor Frequencies

Procedures for flow cytometry staining (including buffers used) were performed as described previously with minor modifications (87). Briefly, 100 μl of MNCs at 1 × 10^7^ cells/ml were stained with anti-porcine CD21-PE (clone BB6-11C9.6, Southern Biotech) and anti-porcine CD2 (clone MSA4, VMRD) monoclonal Abs (mAbs) to determine B cell subsets (88). To determine expression of α4 integrin, β7 integrin and CCR10, cells were stained with porcine cross-reactive anti-human α4 integrin (clone HP2/1, Abcam, Cambridge, MA), anti-mouse β7 integrin (clone FIB27, BD Biosciences), and anti-mouse CCR10 (clone 248918, R&D Systems) mAbs. Additionally, to determine expression of IgA, cells were stained with anti-porcine IgA (clone K61 1B4, Bio-Rad) mAb. After washing, cells were stained with appropriate secondary antibodies. For intracellular CD79β staining, stained cells were permeabilized with Cytofix/Cytoperm (BD Biosciences), washed with Perm/Wash Buffer (BD Biosciences), and stained with porcine cross-reactive anti-mouse CD79β-FITC Ab (clone AT1072, Bio-Rad) mAb. Additionally, CD4^+^ (anti-porcine CD4, clone 74-12-4, Southern Biotech) and CD8^+^ (anti-porcine CD8, clone 76-2-11, Southern Biotech) T cells were assessed within the CD3^+^ (anti-porcine CD3, clone PPT3, Southern Biotech) MNC population (T lymphocytes). Appropriate isotype matched control antibodies were included. Acquisition of 50,000 events and analyses were done using the Accuri C6 flow cytometer (BD Biosciences, San Jose, CA, USA). The gating strategy for T and B cell phenotypes are depicted in Figures S1, S2.

### Statistics

PEDV RNA shedding titers, fecal consistency scores, normalized weights, frequencies of blood MNC populations in flow cytometry, mean concentrations of serum cytokines, PEDV IgA and IgG ASCs, log-transformed PEDV IgA, IgG and VN Ab titers and PEDV Ab^+^ cells in the MG were analyzed by a two-way analysis of variance (ANOVA-general linear model), followed by Bonferroni's posttest. NK cell activity in blood and PEDV IgA and IgG ASCs in MG, spleen, MLN, and ileum tissues were compared among groups with the Mann-Whitney (non-parametric) test. The log-rank (Mantel-Cox) test was used for comparison of survival curves amongst treatment groups. Statistical significance was assessed at *P* ≤ 0.05 for all comparisons. All statistical analyses were performed with GraphPad Prism 5 (GraphPad Software, Inc., CA).

## Ethics Statement

Studies were approved by the Institutional Animal Care and Use Committee (IACUC) and performed on gestating and lactating gilts and piglets aged 0–5 weeks, in accordance with USDA and OSU IACUC guidelines.

## Author Contributions

LS, KL, and SL contributed conception and design of the study. SL conducted the experiments, analyzed the data, and wrote the manuscript. LS and AG supervised the work and contributed critical analysis to the results. FP, MA, AB, and AVG assisted with daily animal work, collected and processed samples and conducted RT-qPCR experiments and analysis. MA and SL conducted IHC experiments and analysis. All authors contributed to manuscript revision, read, and approved the submitted version.

### Conflict of Interest Statement

The authors declare that the research was conducted in the absence of any commercial or financial relationships that could be construed as a potential conflict of interest.
