# Supplementary material for: Stage of Gestation at Porcine Epidemic Diarrhea Virus Infection of Pregnant Swine Impacts Maternal Immunity and Lactogenic Immune Protection of Neonatal Suckling Piglets
Source: Front Immunol. 2019 Apr 24;10:727. doi: 10.3389/fimmu.2019.00727 (PMC6491507; doi:10.3389/fimmu.2019.00727)
Supplement: Supplementary file 1 [file Data_Sheet_1.docx]

Supplementary Material

**Stage of Gestation at Porcine Epidemic Diarrhea Virus Infection of Pregnant Swine Impacts Maternal Immunity and Lactogenic Immune Protection of Neonatal Suckling Piglets**

**Stephanie N. Langel, Francine C. Paim, Moyasar, A. Alhamo, Alexandra Buckley, Albert Van Geelen, Kelly M. Lager, Anastasia N. Vlasova, Linda J. Saif^*^**

*** Correspondence:** Linda J. Saif: [saif.2@osu.edu](mailto:saif.2@osu.edu)


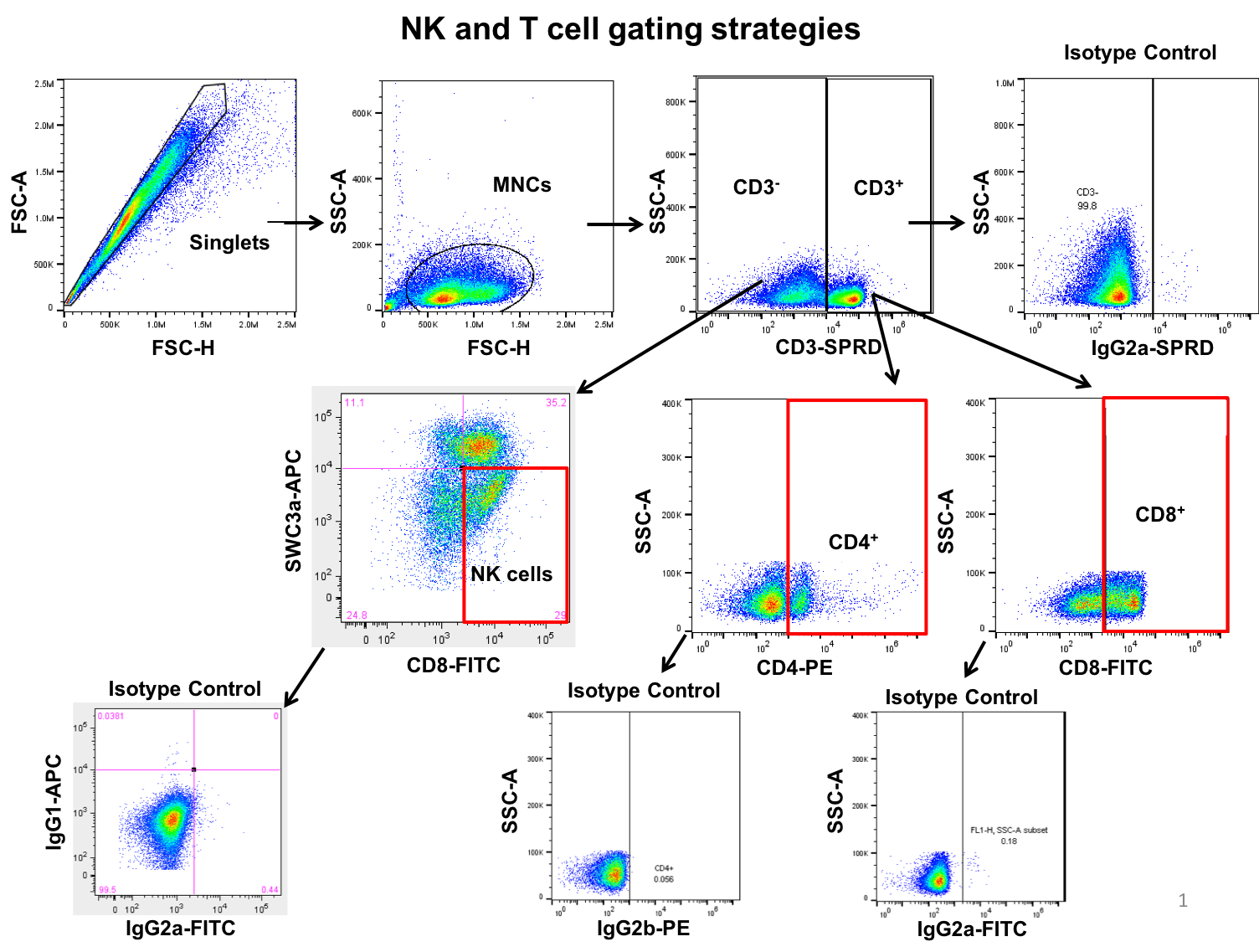


**Figure S1.** Natural killer (NK) and T cell gating strategies. Acquisition of 50,000 events and analyses were done using the Accuri C6 flow cytometer (BD Biosciences, San Jose, CA, USA).

**
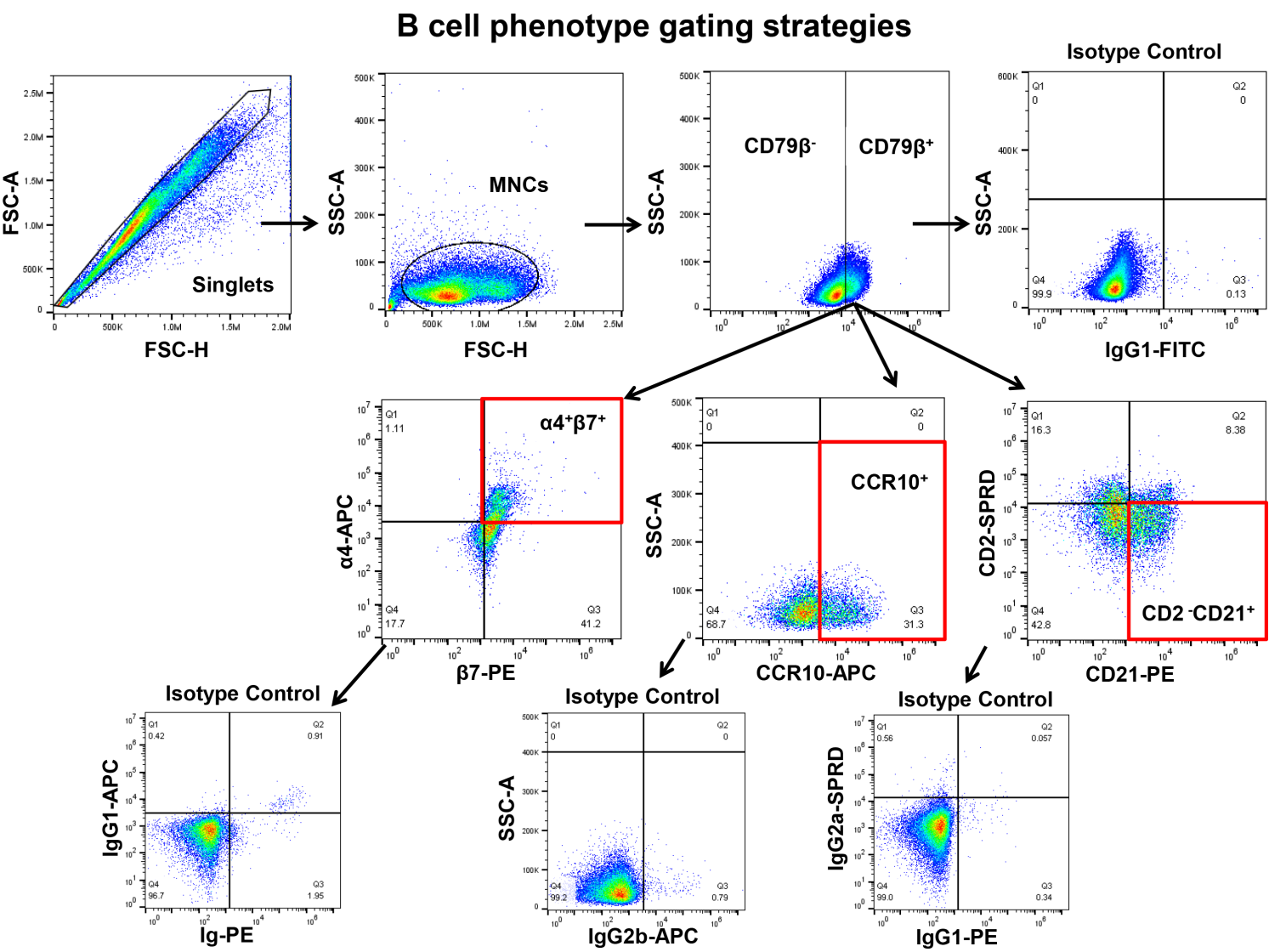
**

**Figure S2.** B cell gating strategies. Acquisition of 50,000 events and analyses were done using the Accuri C6 flow cytometer (BD Biosciences, San Jose, CA, USA).


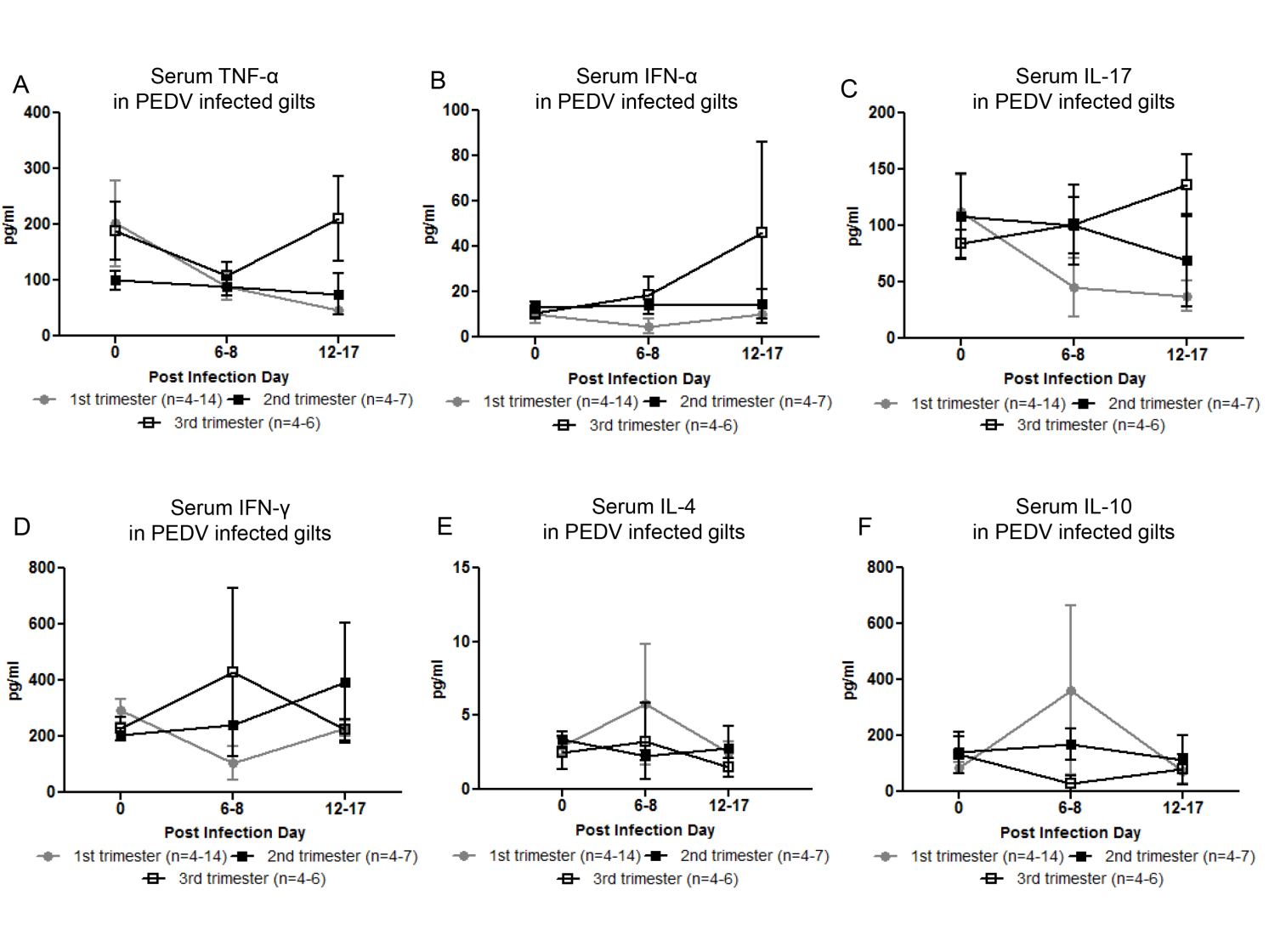


**Figure S3.** Comparison of serum cytokine concentrations (mean ± SEM) in first, second and third trimester PEDV-infected gilts at post infection day (PID) 0, 6-8 and 12-17. **(A)** tumor necrosis factor (TNF)-α **(B)** interferon (IFN)-α **(C)** interleukin (IL)-17 **(D)** IFN-γ **(E)** IL-4 **(F)** IL-10. Statistical analysis was performed using the two-way ANOVA with repeated measures and Bonferroni’s correction for multiple comparisons.


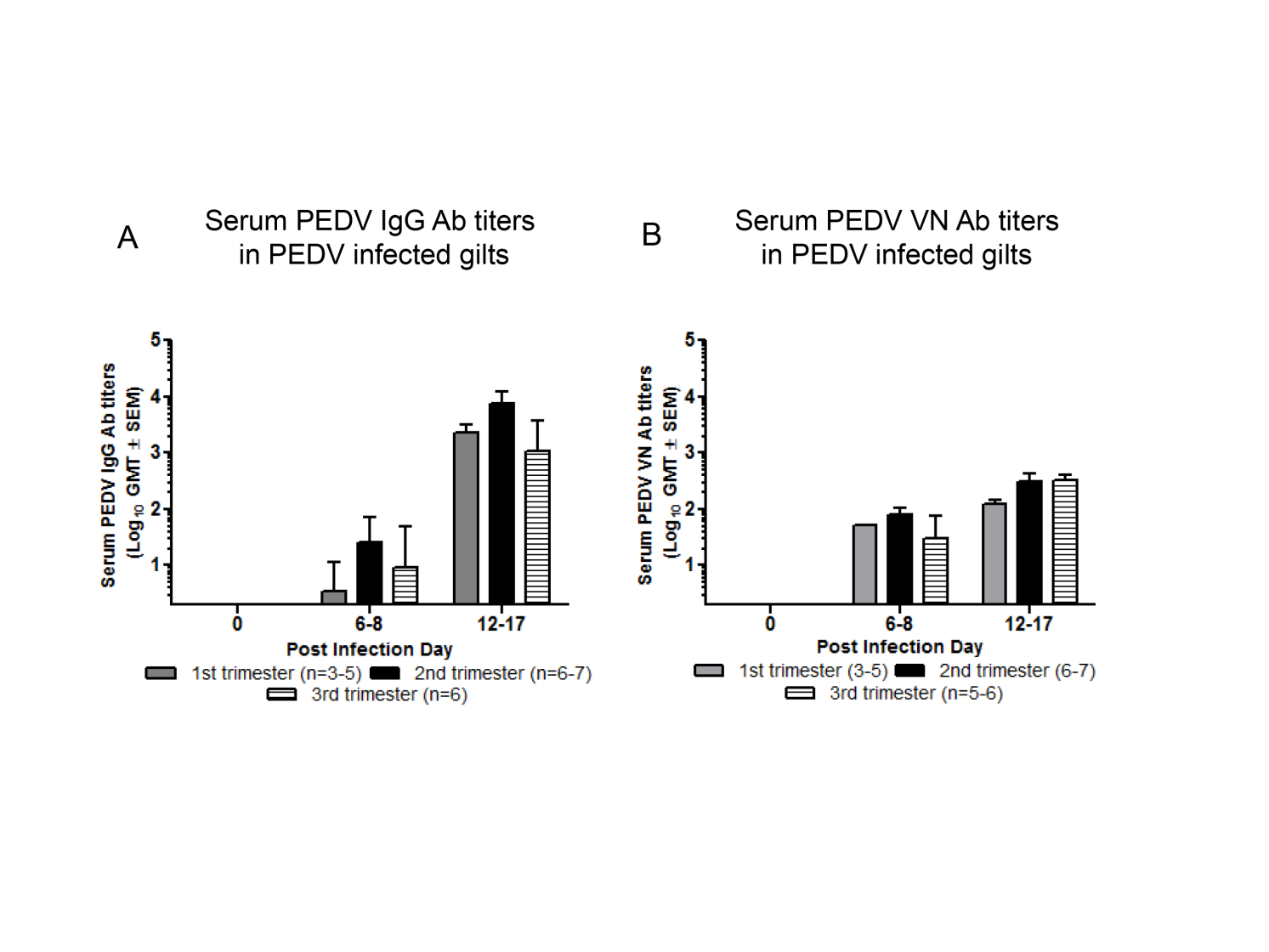


**Figure S4.** Comparison of circulating PEDV IgG and virus neutralizing (VN) antibodies (Abs) post PEDV infection. **(A)** Serum PEDV IgG was determined by ELISA while **(B)** serum PEDV VN Ab responses were determined by VN Ab assay. Gilts were sampled at post infection day (PID) 0, 6-8 and 12-17 (mean ± SEM). Statistical analysis was performed using the two-way ANOVA with repeated measures and Bonferroni’s correction for multiple comparisons.

**

**

**Figure S5**. Comparison of the number of viable piglets among treatment groups at post challenge day (PCD). Data are (mean ± SEM). Statistical analysis was performed using a one-way ANOVA.


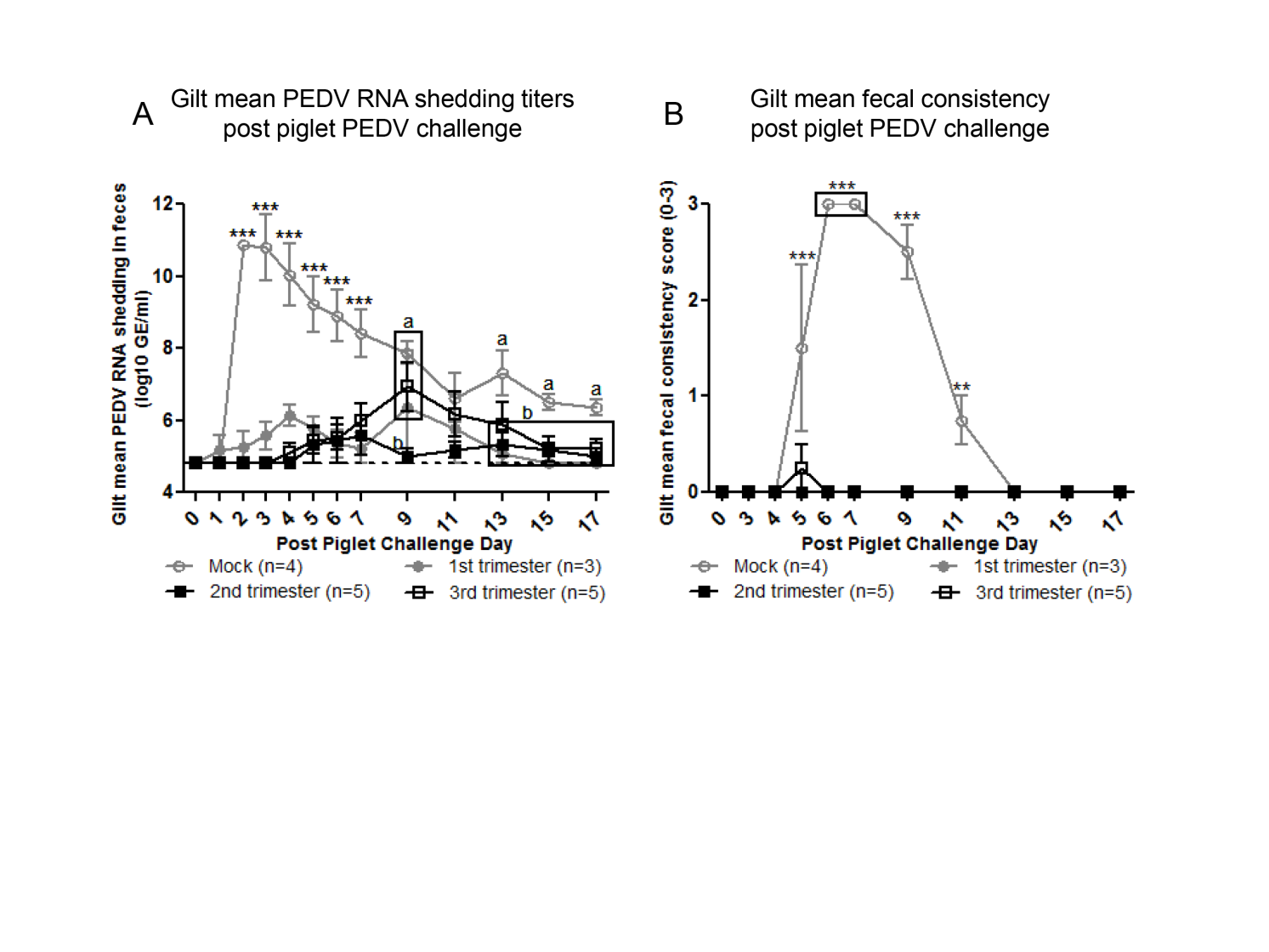


**Figure S6.** **(A)** Gilt PEDV RNA shedding titers were determined by real time quantitative polymerase chain reaction (qRT-PCR) and expressed as log_10_ copies/ml. **(B)** Gilt diarrhea was determined by fecal consistency score >1 (fecal consistency was scored as follows: 0, normal; 1, pasty/semiliquid; 2, liquid; 3, watery). Different letters and/or asterisks indicate significant differences among treatment groups at the same time point (mean ± SEM). Statistical analysis was performed using the two-way ANOVA with repeated measures and Bonferroni’s correction for multiple comparisons. **P* < 0.05 ***P* < 0.01 ****P* < 0.001.


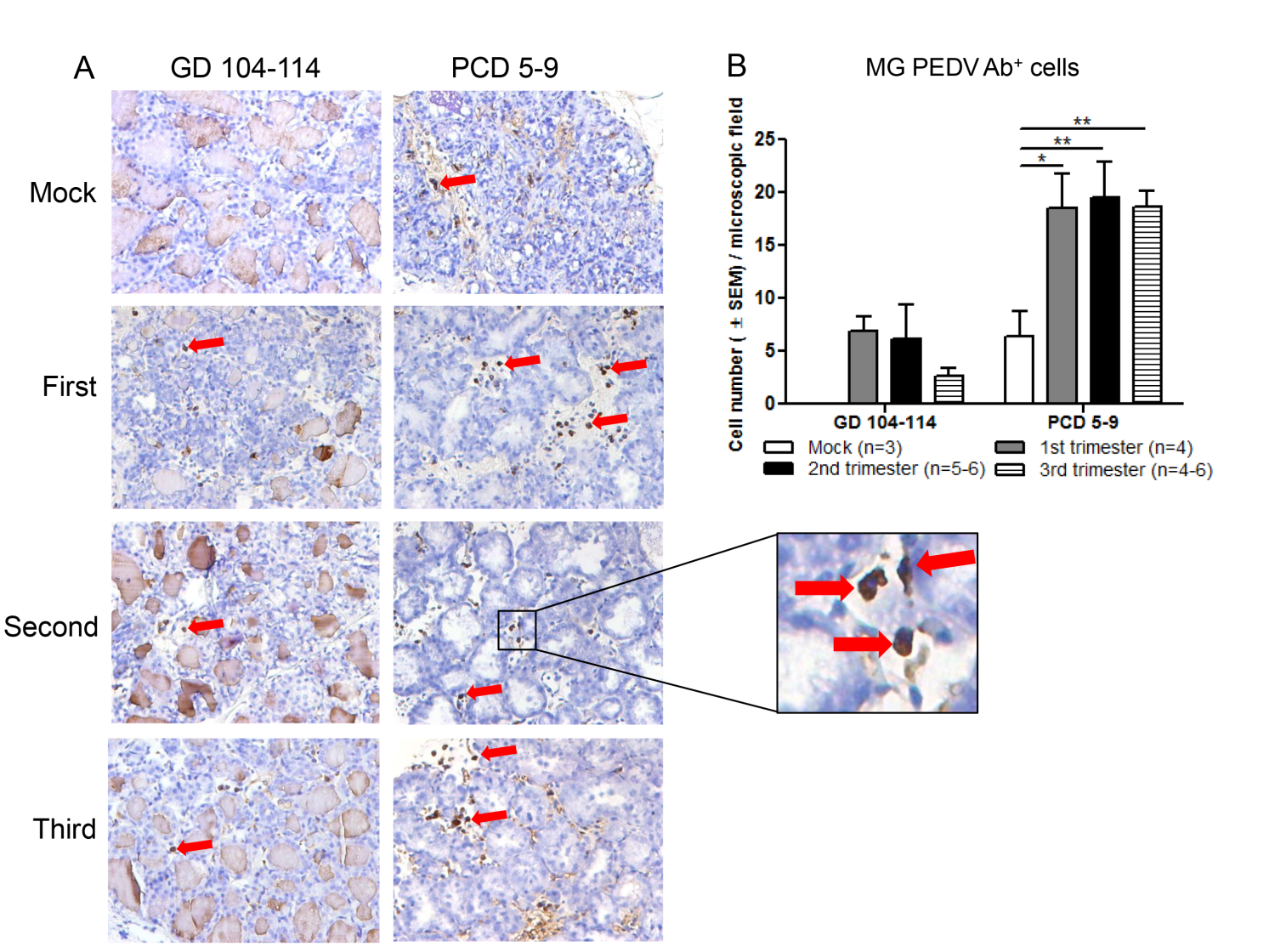


**Figure S7.** PEDV antibody (Ab)^+^ cells in the mammary gland (MG) increase post PEDV challenge. **(A)** Evaluation of PEDV Ab^+^ cells in the MG at gestation day (GD) 104-114 and post challenge day (PCD) 5-9 in first, second and third trimester PEDV-infected and mock gilts by PEDV viral suspension sandwich immunohistochemistry (IHC) method and hematoxylin and eosin (H&E) staining (30×). Right: enlarged view of MG tissue where red arrows indicate PEDV Ab^+^ cells. **(B)** Cells were quantified by averaging PEDV Ab^+^ cells from 3-6 microscope fields (30×) from different areas of the MG for each sample time point (GD 104-114 and PCD 5-9) from first, second and third PEDV-infected or mock gilts. Statistical analysis was performed using the one-way ANOVA and Dunn’s posttest for multiple comparisons. **P* < 0.05 ***P* < 0.01 ****P* < 0.001.


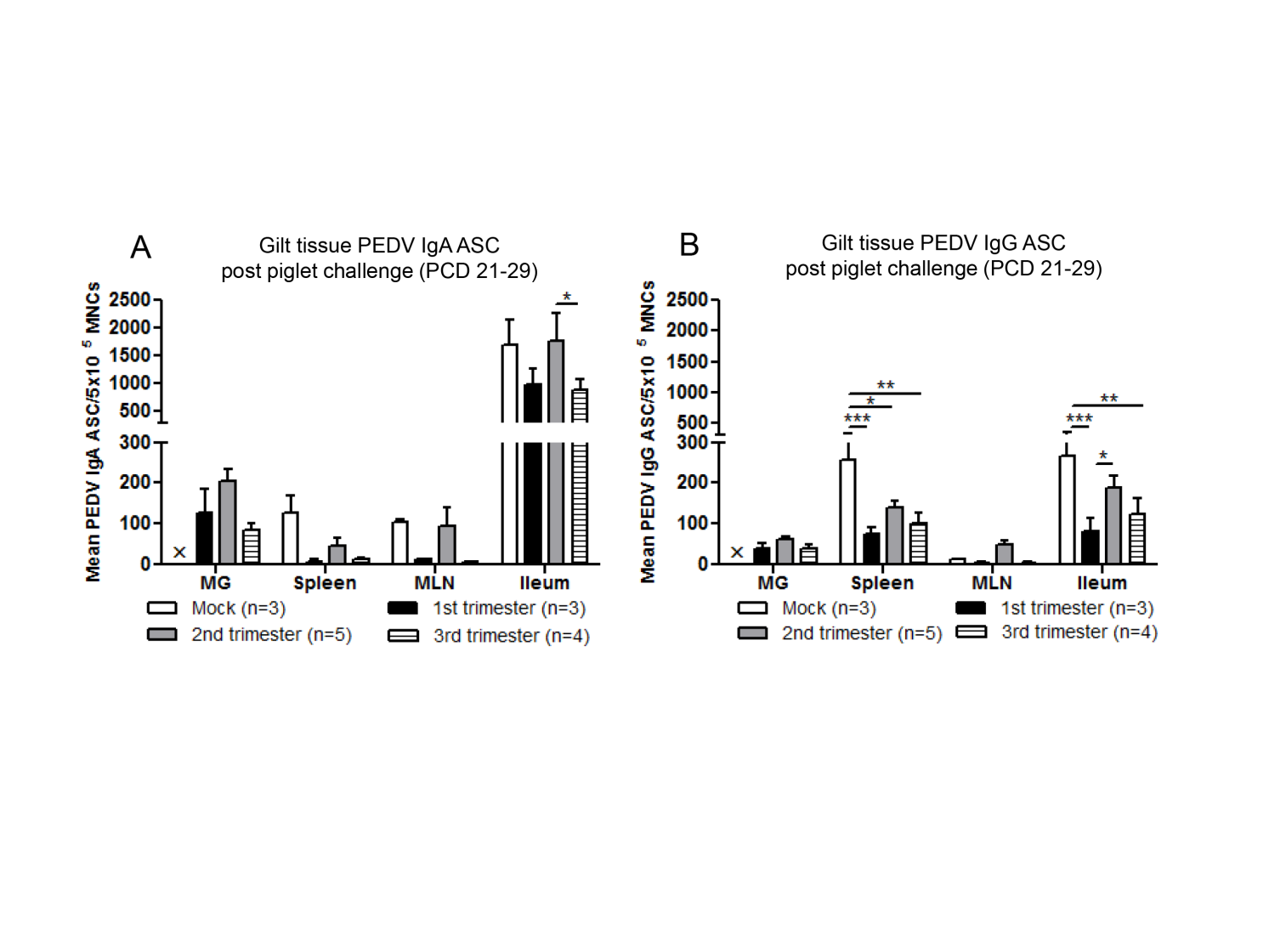


**Figure S8. (A,B)** Comparison of IgA and IgG PEDV antibody secreting cells (ASCs) in mammary gland (MG), spleen, mesenteric lymph node (MLN) and ileum tissue at post challenge day (PCD) 21-29. Asterisks indicate significant differences among treatment groups at the same time point (mean ± SEM). Statistical analysis was performed using the two-way ANOVA with repeated measures and Bonferroni’s correction for multiple comparisons. **P* < 0.05 ***P* < 0.01 ****P* < 0.001.
